# Plasma Extracellular Vesicle miRNAs Can Identify Lung Cancer, Current Smoking Status, and Stable COPD

**DOI:** 10.3390/ijms22115803

**Published:** 2021-05-28

**Authors:** Hannah E. O’Farrell, Rayleen V. Bowman, Kwun M. Fong, Ian A. Yang

**Affiliations:** The University of Queensland Thoracic Research Centre, The Prince Charles Hospital, Chermside, QLD 4032, Australia; rayleen.bowman@health.qld.gov.au (R.V.B.); kwun.fong@health.qld.gov.au (K.M.F.); Ian.Yang@health.qld.gov.au (I.A.Y.)

**Keywords:** extracellular vesicles, miRNAs, plasma, lung cancer, chronic obstructive pulmonary disease

## Abstract

Lung cancer remains the leading cause of cancer related mortality worldwide. We aimed to test whether a simple blood biomarker (extracellular vesicle miRNAs) can discriminate between cases with and without lung cancer. Methods: plasma extracellular vesicles (EVs) were isolated from four cohorts (n = 20 in each): healthy non-smokers, healthy smokers, lung cancer, and stable COPD participants. EV miRNA expression was evaluated using the miRCURY LNA miRNA Serum/Plasma assay for 179 specific targets. Significantly dysregulated miRNAs were assessed for discriminatory power using ROC curve analysis. Results: 15 miRNAs were differentially expressed between lung cancer and healthy non-smoking participants, with the greatest single miRNA being miR-205-5p (AUC 0.850), improving to AUC 0.993 in combination with miR-199a-5p. Moreover, 26 miRNAs were significantly dysregulated between lung cancer and healthy smoking participants, with the greatest single miRNA being miR-497-5p (AUC 0.873), improving to AUC 0.953 in combination with miR-22-5p; 14 miRNAs were significantly dysregulated between lung cancer and stable COPD participants, with the greatest single miRNA being miR-27a-3p (AUC 0.803), with two other miRNAs (miR-106b-3p and miR-361-5p) further improving discriminatory power (AUC 0.870). Conclusion: this case control study suggests miRNAs in EVs from plasma holds key biological information specific for lung cancer and warrants further prospective assessment.

## 1. Introduction

Due to the lack of early diagnosis and effective treatments, lung cancer remains one of the most fatal forms of cancer; therefore, diagnosis at an earlier, more curable stage of disease, has been the focus of worldwide efforts to reduce associated mortality. There is a large body of evidence describing the pathological link between chronic obstructive pulmonary disease (COPD)—the third leading cause of death worldwide [1,2]—and lung cancer—the leading cause of cancer-related mortality [3], beyond their common etiologies. While tobacco smoking is the main risk factor for both lung cancer and COPD, only 10–20% of smokers develop COPD [4], and conversely 10–15% of individuals with lung cancer are non-smokers [5]. Additionally, lung cancer is five times more likely to occur in smokers with airflow obstruction than in those with normal lung function [6], with the annual incidence of lung cancer arising in individuals with COPD being reported as 0.8–1.2% [7]. Screening and monitoring of individuals with COPD to identify early stage lung cancer has been suggested as a potential strategy to reduce lung cancer mortality [8]. As part of this approach, the search for novel diagnostic biomarkers of serious lung diseases has been under intense investigation. 

microRNAs (miRNAs) are short non-coding RNAs (19–22 nucleotides) that regulate gene expression post-transcriptionally through either degradation of target mRNA or translation inhibition [9]. Due to the broad range of target genes, miRNAs are involved in regulating a number of key physiological processes including apoptosis, DNA repair, cell metabolism, and the initiation and progression of pathogenic processes, leading to further inflammation and tumourigenesis [10,11,12]. miRNAs have been shown to be highly stable in a variety of body fluids, in particularly plasma [13,14,15]. Studies have shown that, in cells, miRNAs have an estimated half-life of 8 hours [15], unlike mRNA which only have a half-life of several minutes [16]. 

Nanosized lipid bilayer membrane vesicles, known as extracellular vesicles (EVs), have been identified as an attractive source of biomarkers for lung disease. This is due to their key role in intercellular communication, through bioactive cargo (e.g., DNA, RNA, miRNA, proteins) exchange with recipient cells [17]. This bioactive cargo can contain disease specific molecular information, reflecting their cellular origin. Specific miRNAs have been demonstrated to be selectively exported into EVs, while others are excluded [18], altering recipient cells biological processes and overall disease pathophysiology [19]. Given the stability of plasma miRNAs and transportability of vesicles, their accessibility through minimally invasive methods (i.e., blood tests) [17,20,21] makes them ideal for biomarker studies. 

In this study, we aimed to identify potential diagnostic EV miRNA biomarkers that can discriminate between cases of lung cancer and COPD, as well as separate these disease states from healthy smokers and healthy non-smoking participants. Plasma EV miRNAs with discriminatory power to distinguish lung cancer from COPD and from smokers without lung disease could have significant translational application as a screening tool for lung cancer case finding in populations at risk.

## 2. Results

### 2.1. Clinical Characteristics of the Four Case Groups

EVs were isolated from plasma, extracted for RNA and assessed for miRNA expression from 20 healthy participants who had never smoked, 20 healthy smokers, 20 participants with a smoking history and diagnosis of non-small cell lung cancer, and 20 participants with clinically stable COPD. Clinical characteristics are shown in Table 1. Each of the four groups contained similar numbers of males and females, although the healthy controls were on average 10 years younger than cases in the other three groups (*p* < 0.0001). The majority of the “healthy” smoker group were current smokers, whereas most of the cases of diagnosed lung disease (lung cancer or COPD) were former smokers (see Table 1), but there was no significant difference in cumulative pack years between the three groups. The majority of lung cancer cases were TNM stage I or II. Most of the COPD cases had GOLD-classified moderate to severe disease.

### 2.2. Characterization of EVs from Healthy Non-Smoking, Healthy Smoking, Lung Cancer, and Stable COPD Participants Plasma

An aliquot of EVs isolated from plasma of two healthy non-smokers, two healthy smokers and two lung cancer participants were characterized using western blotting, while one healthy non-smoker case underwent nanoparticle tracking analysis (NTA). 

**Figure 1:**
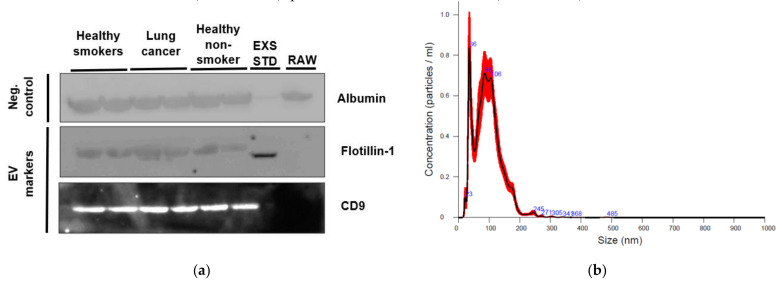
Plasma derived EV characterization. (**a**) Western blot of negative control marker Albumin and EV markers Flotillin-1 and CD9 presence or absence in participant groups (healthy smokers, lung cancer, healthy non-smokers), as well as lyophilized exosome standard from plasma (EXS STD) and raw plasma (RAW). (**b**) Plasma EV particle concentration and size from a healthy non-smoking participant using nanoparticle tracking analysis (NTA).

Established “EV-markers” Flotillin-1 and CD9 were identified by western blotting in plasma EVs from the cases, but were absent from the corresponding raw plasma samples (Figure 1). An exosome standard from human plasma used as a positive control was positive for Flotillin-1 (but not CD9) in the EV samples. Albumin, the most abundant protein in plasma [22], was present in the raw plasma samples on western blots as expected, and showed a faint band in the exosome standard, but was also identified in plasma EVs, so may not be a suitable “negative” control marker.

Characterization using NTA was performed on one healthy non-smoker participant’s plasma EVs. The concentration of EV particles was 7.02 × 10^8^ ± 7.26 × 10^7^ particles/mL (mean ± 1SD), particle size was 96.6 ± 1.6 nm (mean ± 1SD), and mode was 76 nm.

### 2.3. Plasma-Derived EV miRNA Profiling Identified Significantly Dysregulated miRNAs Specific for Select Cohorts

**Figure 2:**
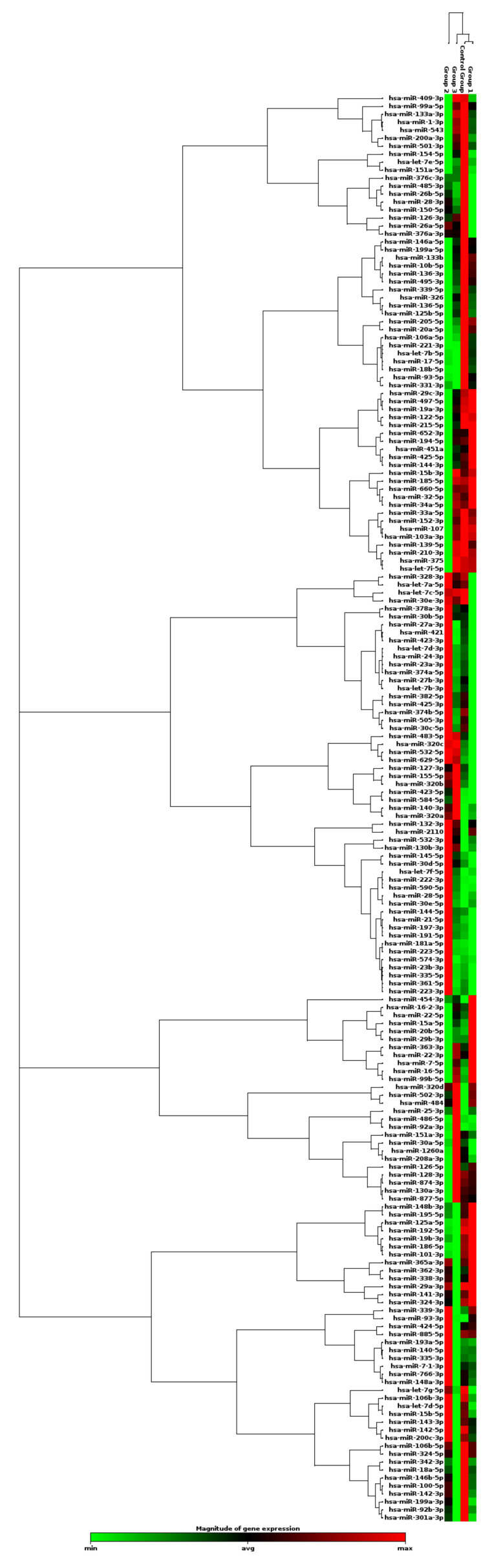
Clustergram indicating the magnitude of miRNA expression (green = minimal, red = maximal). Clustering is grouped by cohort (control group = healthy non-smokers, group 1 = healthy smokers, group 2 = lung cancer and group 3 = stable COPD).

Candidate plasma-derived EV miRNAs were investigated by qPCR to identify those that could discriminate between lung disease cohorts (lung cancer and stable COPD), healthy smokers, and healthy non-smokers. Testing for 179 miRNAs specific for human serum and plasma (including internal controls) was performed using QIAGEN’s miRCURY LNA miRNA Serum/Plasma Focus PCR panels. A clustergram of miRNA expression across all cohorts is shown in Figure 2.

As detailed in Table 2, the miRNA expression from plasma EVs identified several miRNAs whose expression differed between the four cohorts.

Two plasma EV miRNAs were expressed at significantly higher levels in lung cancer participants than in healthy non-smokers and expression of 13 miRNAs was significantly lower in the lung cancer participants.

Additionally, lung cancer participants compared to the healthy smoker’s cohort identified 14 significantly over- and 12 significantly under-expressed miRNAs. 

Further, lung cancer participants compared to stable COPD patients identified 6 miRNAs that were significantly under-expressed and eight miRNAs that were significantly over-expressed. 

**Figure 3:**
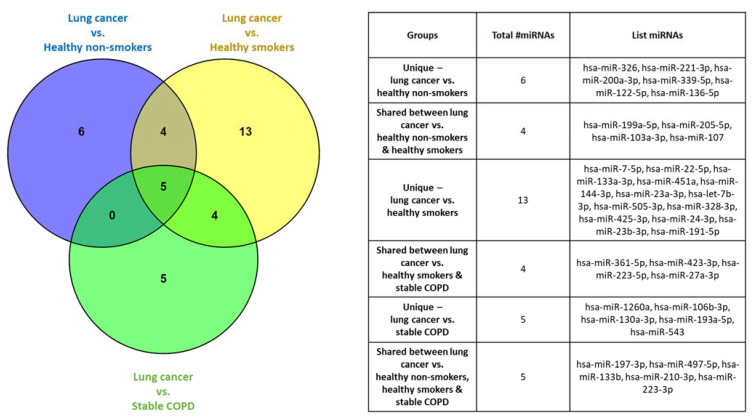
Venn diagram listing the dysregulated miRNAs unique and common for lung cancer participants and comparator groups including healthy non-smokers, healthy smokers, and stable COPD participants. Venn diagram created using Venny 2.1 software [23].

A total of five miRNAs were consistently identified as significantly dysregulated in lung cancer participants compared to all three other cohorts (miR-197-3p, miR-223-3p, miR-133b, miR-497-5p and miR-210-3p) (Figure 3). Six miRNAs were unique in discriminating lung cancer participants from healthy non-smokers, 13 miRNAs from healthy smokers and five miRNAs from stable COPD participants (Figure 3).

Further, candidate miRNA-regulated genes were identified based on the miRNAs expression in lung cancer participants compared to healthy non-smokers, healthy smokers, and stable COPD patients (full list detailed in Table S1).

miRNA-regulated gene ZXDC was unique to lung cancer participants compared to healthy non-smokers, targeted by two over-expressed miRNAs. NFIB was unique to lung cancer participants compared to healthy smokers, targeted by five overexpressed miRNAs. While CLCN5 was unique to lung cancer participants compared to stable COPD participants, targeted by four under-expressed miRNAs.

### 2.4. Correlations Identified between Significantly Dysregulated miRNAs and Clinical Characteristics

Significant differences and correlations were assessed between the identified miRNAs and appropriate clinical characteristics between lung cancer participants compared to comparator groups (healthy non-smokers, healthy smokers, and stable COPD).

Two plasma EV miRNAs differentially expressed between lung cancer participants and healthy non-smokers were significantly associated with age (Table S2), while four plasma EV miRNAs were significantly associated with gender (Table S3). 

For lung cancer participants, compared to healthy smokers, five plasma EV miRNAs were significantly associated with age (Table S4), while seven plasma EV miRNAs were significantly associated with gender and three miRNAs with smoking history (Table S3). 

For lung cancer participants compared to stable COPD participants, five plasma EV miRNAs were significantly associated with age, two plasma EV miRNAs were associated with pack years (Table S5), while three plasma EV miRNAs were significantly associated with gender (Table S3).

### 2.5. Biomarker Potential of Plasma EV miRNAs Assessed by ROC Curve Analyses

To evaluate the discriminatory power of plasma EV miRNAs for distinguishing lung cancer from healthy non-smoking, healthy smoking and stable COPD groups, ROC curve analysis was performed on select miRNAs that did not have significant associations with clinical characteristics.

### 2.6. Lung Cancer Participants vs. Healthy Non-Smokers

**Figure 4:**
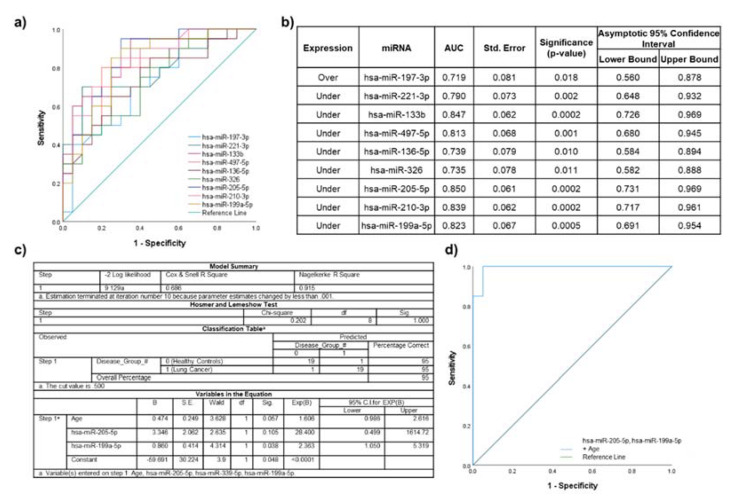
Discriminatory power assessed using ROC curves for the miRNAs that were significantly dysregulated in lung cancer participants compared to healthy non-smoker participants. (**a**) Individual ROC curves for under- and overexpressed miRNAs; (**b**) AUC values, including standard error (Std. Error), significance and 95% confidence intervals; (**c**) binary logistic regression model for miR-205-5p, miR-199a-5p, and significant clinical characteristic (age); (**d**) ROC curve for the logistic regression model.

A total of 15 miRNAs identified in the primary analyses were significantly dysregulated between lung cancer participants compared to healthy non-smoking participants. From Table S2, the expression of six miRNAs was significantly associated with age and/or gender (Table S3). ROC curve analysis was performed individually for the remaining nine significantly over- or under-expressed miRNAs (Figure 4a). The highest AUC for a single miRNA was achieved by miR-205-5p (AUC 0.850; standard error 0.061; 95% CI 0.731-0.969; *p* = 0.0002) (Figure 4b).

The miRNAs that were not significantly correlated with each other or associated with demographic parameters with the highest AUC values were miR-205-5p and miR-199a-5p. These were included in a logistic regression model (Figure 4c) for which ROC curve analysis showed the AUC improved to 0.993 (standard error 0.009; 95% CI 0.974–1.000; *p* < 0.0001) (Figure 4d).

### 2.7. Lung Cancer Participants vs. Healthy Smokers

**Figure 5:**
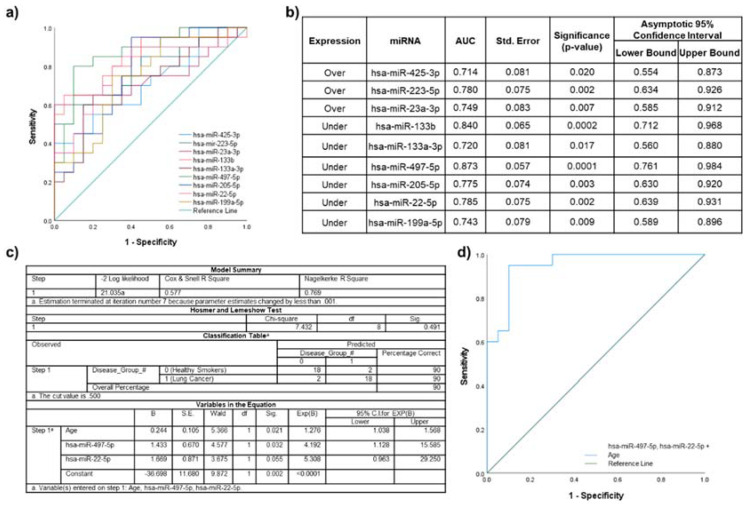
Discriminatory power assessed using ROC curves for the miRNAs that were significantly dysregulated in lung cancer participants compared to healthy smokers. (**a**) Individual ROC curves for under- and overexpressed miRNAs; (**b**) AUC values, including standard error (Std. Error), significance and 95% confidence intervals; (**c**) binary logistic regression model for miR-497-5p, miR-22-5p and significant clinical characteristics (age); and (**d**) ROC curve for the logistic regression model.

A total of 17 of the 26 miRNAs identified in the primary analyses as significantly differentially expressed in plasma EVs between lung cancer participants and healthy smokers were associated with age (Table S4), gender and/or smoking history (Table S3). Individual ROC curve analyses of the remaining nine significantly over- or under-expressed miRNAs are shown in Figure 5a. The highest AUC for a single miRNA was achieved by miR-497-5p (AUC 0.873; standard error 0.057; 95% CI 0.761–0.984; *p* = 0.0001) (Figure 5b).

Based on the highest individual AUC values, and lack of associations with potential confounding factors the combination of miR-497-5p and miR-22-5p was assessed in a logistic regression model including age (Figure 5c). ROC curve analysis of the model (Figure 5d), showed that AUC improved to 0.953 (standard error 0.031; CI 0.892–1.000; *p* < 0.0001).

### 2.8. Lung Cancer Participants vs. Stable COPD Participants

**Figure 6:**
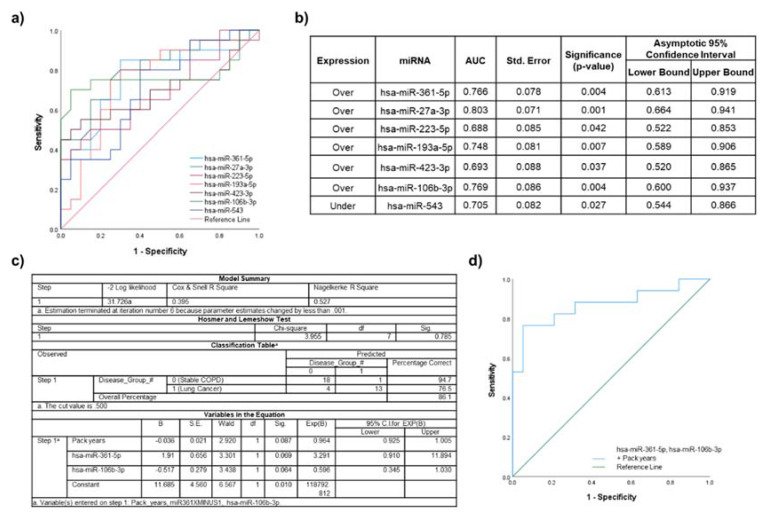
Discriminatory power assessed using ROC curves for the miRNAs that were significantly dysregulated in lung cancer participants compared to stable COPD participants. (**a**) Individual ROC curves for under- and overexpressed miRNAs; (**b**) AUC values, including standard error (Std. Error), significance, and 95% confidence intervals; (**c**) binary logistic regression model for miR-106b-3p and miR-361-5p, as well as significant clinical characteristic (pack years); and (**d**) ROC curve for the logistic regression model.

A total of 14 miRNAs identified in the primary analyses were significantly dysregulated between lung cancer patients compared to stable COPD participants. From the correlation analysis with clinical characteristics, seven miRNAs were significantly correlated with age or significantly associated with gender, smoking history and/or pack years (Tables S3 and S5). ROC curve analysis was performed for the remaining seven significantly dysregulated individual over- and under-expressed miRNAs (Figure 6a). The highest AUC for a single miRNA was achieved with miR-27a-3p (AUC 0.803; standard error 0.071; 95% CI 0.664–0.941; *p* = 0.001) (Figure 6b).

Combination of miR-27a-3p with another with the second highest individual AUC value could not be achieved, as miR-27a-3p significantly correlated with all of the remaining miRNAs. The second highest AUC was achieved by miR-106b-3p, which could be combined with miR-361-5p, as no significant correlations identified with each other or clinical characteristics (with the exception of pack years being significantly different between the two patient cohorts), and they were therefore further assessed in a logistic regression model (Figure 6c). The model was then analyzed using ROC curve analysis (Figure 6d), with the AUC improving to 0.870 (standard error 0.064; CI 0.744–0.996; *p* = 0.0002). 

### 2.9. Biological Pathways Associated with miRNAs Differentially Expressed between Lung Cancer Participants and Healthy Non-Smoking Participants

KEGG pathway analysis was used to identify significantly affected pathways from the candidate miRNAs that were significantly dysregulated in the primary analysis in the lung cancer participants compared to the healthy non-smoking participants (Table 3).

A total of 15 significantly dysregulated miRNAs that were identified in the lung cancer cohort compared to the healthy non-smoker’s cohort were enriched in three different KEGG pathways. The ‘proteoglycans in cancer’ (hsa05205) interaction was the most significant KEGG pathway enriched for the most miRNAs, with 32 enriched target genes, as well as the most genes validated by the miRNA-regulated gene targets GeneGlobe analysis. 

## 3. Discussion

### 3.1. Main Results

In this study, we verified the presence of EVs isolated from plasma and identified significantly dysregulated EV miRNAs that can discriminate between groups of lung cancer cases compared to healthy non-smokers, healthy smokers and stable COPD cases. These EV miRNAs and or signatures specific to disease states have translational application as potential biomarkers with their strong diagnostic discrimination power evaluated using ROC curve analysis. Further, KEGG pathway analysis based on EV miRNAs with discriminatory power between case groups indicates their involvement in disease-specific and biologically relevant pathways.

### 3.2. Lung Disease Biomarker Potential of Plasma EV miRNAs 

There is increasing evidence to support the biomarker potential of miRNAs for diagnosis of disease. It has previously been reported that in plasma, miRNAs are concentrated in the bioactive cargo of EVs [24], which (due to their phospholipid bilayer) are highly stable in circulating bodily fluids. EV cargo reflects the physiological state and microenvironment of the cells of origin and in disease states circulating EVs contain an array of disease associated biomolecules [25]. 

Recent studies in various lung diseases show that certain miRNAs are differentially enriched in EVs, and that these can alter biological processes in recipient cells, thereby reflecting disease pathophysiology [19].

It is known that tobacco smoking is one of the main etiological risk factors for lung cancer and COPD development [26]. Therefore, when identifying possible diagnostic biomarkers, clinically relevant controls need to be evaluated, which in the case of lung cancer and COPD, includes smokers without these diseases.

#### 3.2.1. Lung Cancer Participants vs. Healthy Non-Smokers

Our results identified miR-205-5p as the top under-expressed plasma EV miRNA, with no association identified between age and gender, with solid discriminatory power (AUC 0.850) in distinguishing lung cancer from healthy non-smokers. This result is not concordant with previous reports and literature, in fact miR-205-5p has been extensively reported as being significantly overexpressed lung cancer [27], promoting metastasis and cellular invasion through an epithelial phenotype, along with increased E-cadherin and reduced fibronectin [28].

Explanations for this anomaly may include the small sample sizes of both cohorts and therefore validation of this result is required in larger, independent cohorts. Additionally, this result may have occurred due to mechanisms involved in selective miRNA packaging into EVs, a process that is not random, as certain miRNAs can be exported into EVs, while others are excluded [29], which suggests that their effect can be pathogenic for specific lung diseases [30] and not just a ‘bystander’. The mechanisms behind EV miRNA packaging are complex and still under investigation. Previous reports have suggested that specific miRNA enriched EVs can exert anti-tumorigenic effects to nearby cells [31]. A cell surface heparin sulfate proteoglycan known as syndecan-1 has been reported to function in cancer cell signaling and exosomes from cells expressing this proteoglycan have shown to contain a miRNA (miR-485) that was both upregulated in A549 cells [32], while another study reported this miRNA as downregulated in breast cancer tissue [33]. Additionally, miRNA profiling of plasma fractions revealed that miR-205 expression increased in tumor specific EVs of patients with squamous cell carcinoma [27,34]; therefore, the decreased expression observed in our primarily adenocarcinoma NSCLC lung cancer participants may be expected [35]. Another alternative explanation may be that signaling molecules produced by tumor cells may downregulate the expression of miRNA from normal tissue, suggesting that miRNAs from non-tumor cells may have diagnostic significance [36].

The discriminatory power for lung cancer participants compared to healthy non-smokers improved with the combination of miR-205-5p and miR-199a-5p (AUC 0.993). Previous studies have reported that miR-205-5p, in combination with other miRNAs, as a validated plasma miRNA signature for lung cancer early detection [37], as well as a differentiating squamous cell carcinoma from adenocarcinoma [38]. For miR-199a-5p, this miRNAs dysregulation is concordant with previous studies, being significantly under-expressed in lung cancer patient’s plasma and tissue [39,40]. The under-expression of miR-199a-5p in the lung cancer cohort [41], has also been demonstrated in adenocarcinoma patients and associated with a high risk for disease progression [41].

Overall these identified plasma EV miRNAs have shown to have strong discriminatory power for lung cancer patients and warrant further validation in a larger independent cohort.

#### 3.2.2. Lung Disease Participant Cohorts 

From our results, significantly dysregulated miRNA, miR-497-5p, may be a potential biomarker candidate for the early detection of lung cancer, as it was identified as being significantly under-expressed in the lung cancer participants compared to healthy non-smokers (AUC 0.813), healthy smokers (AUC 0.873), and stable COPD participants (significantly dysregulated, but not applicable for discriminatory power assessment due to correlations with clinical characteristics). This is in support of previous studies that have shown that in tissue and plasma, miR-497-5p is downregulated in a number of different cancers [42,43,44,45,46], and in relation to NSCLC, this miRNAs has been suggested to function as a tumor suppressor [47,48], as well as related to disease progression, TNM stage and distant metastases [49].

The top identified miRNAs that were significantly dysregulated in the lung cancer cohort compared to stable COPD participants included the over-expression of miR-27a-3p and miR-106b-3p. These findings are concordant with previous studies, with miR-27a-3p being reported as an oncogene with high expression reported in a number of different cancers, including lung cancer [50,51,52]. This miRNAs over-expression has also been suggested to be involved in chemotherapy resistance, as well as disruption of TP53/miR-27a/EGFR pathway, promoting increased cell proliferation and tumorigenesis [53]. Further plasma exosomal miR-27a has been reported as a novel diagnostic and prognostic biomarker for colorectal cancer [54], highlighting this plasma EV miRNAs translational application as a potential biomarker for lung cancer.

In relation to miR-106b, a recent study by Sun et al. reported that serum exosomal miR-106b was significantly higher in lung cancer participants, promoting metastasis through targeting phosphatase and tensin homolog (PTEN) [55]. In regards to COPD, miR-106b has been reported as being significantly under-expressed [56] as well as negatively correlating with disease severity [57]. Overall, results from this study support that these identified plasma EV miRNAs have discriminatory potential to distinguish between lung cancer and COPD, and therefore warrant further investigation as clinically applicable diagnostic biomarkers.

### 3.3. Identified miRNA-Regulated Target Genes in Each Patient Cohort and Their Involvement in Relevant Biological Pathways 

Identifying which miRNAs regulate a given gene set or pathway is a key question to address in functional miRNA studies. In this study, analysis using DIANA-miRpath (v.3.0) identified specific biological pathways from the combinations of miRNAs significantly dysregulated between lung cancer and healthy non-smoker participants.

KEGG pathway analysis identified that the function of these miRNAs were enriched in the proteoglycans in cancer pathway. This is concordant with a recent study by Wu et al. who investigated EV miRNA expression in NSCLC patients and non-smoking controls, and identified that the most prominent pathways enriched in NSCLC EV miRNA signatures was also the proteoglycans pathway, as well as fatty acid biosynthesis [58]. Further, it has been reported that heparin sulfate proteoglycans is a functionally relevant and targetable entry pathway for cancer cell exosomes [59].

Our results further support that the dysregulated plasma EV miRNAs identified in lung cancer participants target specific genes involved in significant lung cancer biological pathways and, therefore, make strong biomarker candidates for lung cancer diagnosis and disease differentiation.

### 3.4. Limitations

The limitations of this study arise from retrospective case control design introducing difficulties with confounding biases. While case control studies for novel biomarkers allow for the comparison of individuals with the outcome of interest (lung cancer) versus without the outcome (no lung cancer), unbalanced confounders, such as age and gender, may be encountered [60,61]. In this study, there was a significant difference in age between lung disease groups and the controls was observed. A larger study with age and gender matched selection of cases and controls would possibly overcome these confounding biases. To adjust for this in the analyses, demographic, and other possible confounding factors were included in the logistic regression models.

Secondly, the relatively small number of cases in groups introduces multiple comparison issues and statistical bias with limitations of overfitting and over-calling significant results. Further validation of the predictive models is required as well as assessment of whether these models are well calibrated. Additionally, a larger study design would have allowed for implementation of training and test sets. Independent external dataset validation of the significant EV miRNAs and signatures would also have strengthened confidence in the reported results.

In this study, a precipitation-based method for EV isolation was used, with advantages of being cost effective, fast, and yielding high volumes of EVs suitable for downstream miRNA analyses. However it has been shown that precipitation methods can also co-isolate contaminating proteins, which interfere with downstream EV characterization [62]. This was observed in the western blot with the presence of plasma contaminating protein albumin in the EV samples. One recently reported way to overcome this issue and improve EV purity from precipitation methods, is the use of protease K and acidification which preserves the advantages of precipitation based EV isolation and minimizes contamination with non-vesicle miRNA [63].

Other studies have reported different unique EV miRNAs and highlighted their potential as novel biomarker signatures for lung disease development and diagnosis. Discrepancies between these and the miRNAs identified in this study may be due to factors such as different cases, comparator groups, and circulating EV compartment (serum vs. plasma vs. whole blood derivation), as well as differing EV isolation and nucleic acid extraction methods, miRNA technology platforms and bioinformatics analyses. For this study, plasma was selected as it was known to yield EVs reliably and a method of miRNA analysis that could be implemented in a clinical setting was chosen.

## 4. Materials and Methods

### 4.1. Statement of Ethics Approval

Protocols and participant recruitment were approved by the Human Research Ethics Committee for the Metro North Hospital and Health Service (HREC/17/QPCH/54 and LNR/2019/QPCH/52409) and The University of Queensland (2019001147). All participants provided written informed consent. Demographics and clinical data were obtained from medical records at the time of sample collection. Clinical data included smoking history, lung cancer staging using the tumor, node, metastasis (TNM) number staging system, and COPD severity using the global initiative for chronic obstructive lung disease (GOLD) airflow limitation severity classification. A summary of the cohort’s clinical characteristics can be found in Table 1.

### 4.2. Blood Plasma Sample Collection and Processing

Full details of blood processing and downstream methods are provided in the online supplement. Briefly, peripheral blood obtained from 80 participants (20 healthy non-smokers, 20 healthy smokers, 20 participants with tissue diagnosed non-small cell lung cancer, and 20 participants with stable COPD) was processed to separate plasma from the blood cell fraction.

### 4.3. Plasma EV Isolation

Frozen plasma samples were thawed and EVs were isolated using the commercially available miRCURY Exosome Serum/Plasma Kit (QIAGEN, Hilden, Germany) according to the manufacturer’s instructions. Briefly, 600 µL of thawed plasma was incubated with thrombin for defibrination, followed by EV precipitation with 500 µL of the thrombin treated plasma and 0.4 volumes of Precipitation Buffer A. Samples were then incubated at 4 °C for 1 hour and then centrifuged at 500× *g* for 5 min at 20 °C. The supernatant was then separated and the remaining EV pellet was either re-suspended in 200 µL 1X PBS, and stored at −80 °C for downstream characterization, or re-suspended in 700 µL QIAZOL as per the manufacturer’s protocol for RNA purification using the miRNeasy Mini Kit (QIAGEN, Hilden, Germany).

### 4.4. Plasma EV Characterization 

#### 4.4.1. Nanoparticle Tracking Analysis

EVs isolated from plasma and re-suspended in 1X PBS were analyzed by nanoparticle tracking analysis (NTA) using the NanoSight NS300 instrument (Malvern Instruments, Amesbury, UK).

#### 4.4.2. Western Blot of EV Markers

Protein concentration of re-suspended EVs in 1X PBS was assessed using the Pierce BCA Protein Assay Kit (Thermo Fisher Scientific, USA) as per the manufacturer’s instructions, followed by blotting for proteins Albumin, Flotillin-1 and anti-CD9 through SDS–PAGE.

### 4.5. Plasma EV RNA Extraction and Purification

Total RNA was extracted using the miRNeasy Mini Kit (QIAGEN, Hilden, Germany) as per the manufacturer’s instructions.

### 4.6. Plasma EV miRNA Profiling

#### 4.6.1. Reverse Transcription 

Reverse transcription (RT) of total RNA was performed with the miRCURY LNA RT kit (QIAGEN, Hilden, Germany) as per the manufacturer’s instructions, using UniSp6 and cel-39 spike-ins as internal controls to detect the presence of potential inhibitors.

#### 4.6.2. Quantitative PCR Using miRCURY LNA Serum/Plasma Focus PCR Panels

Samples from the RT reaction were prepared with the miRCURY SYBR Green PCR Kit (QIAGEN, Hilden, Germany) and assessed for miRNA gene expression using the miRCURY LNA miRNA Serum/Plasma Focus PCR Panels (QIAGEN, Hilden, Germany) as per the manufacturer’s protocol. Raw Ct values were uploaded onto the QIAGEN data analysis web portal at http://www.giagen.com/geneglobe and normalized using the NormFinder algorithm [64,65,66] with fold change expression calculated using the ΔΔCt method.

### 4.7. miRNA Function Enrichment Analysis

Kyoto Encyclopedia of Genes and Genomes (KEGG) pathway analysis was performed using the online software DIANA-miRPath v.3.0, and miRNA targets were also identified using TargetScan [67].

### 4.8. Statistical Analyses

All statistical analyses were performed using GeneGlobe (http://www.giagen.com/geneglobe), DIANA-miRPath v.3.0 and Statistical Package for Social Science (SPSS V.27.0) (IBM, NY, USA). A p-value of <0.05 (two-tailed) was statistically significant. Full details are available on the online supplement. 

## 5. Conclusions

In conclusion, our results highlight that bioactive cargo (miRNAs) in EVs from plasma holds key biological information specific for lung cancer, with diagnostic biomarker potential, which warrants further investigation of translational application.

Fifteen miRNAs were significantly dysregulated between lung cancer participants and healthy non-smokers, with miR-205-5p resulting in the highest AUC (0.850) for a single miRNA, with a combination of two miRNAs (miR-205-5p and miR-199a-5p) further improving discriminatory power (AUC 0.993). 

Twenty-six miRNAs were significantly dysregulated between lung cancer and healthy smoking participants, with miR-497-5p resulting in the greatest AUC (0.873) for a single miRNA, improving to AUC 0.953 in combination with miR-22-5p.

Fourteen miRNAs were significantly discriminatory between lung cancer and COPD participants, of which miR-27a-3p had the highest AUC (0.803) for a single miRNA, with a combination of two miRNAs (miR-106b-3p and miR-361-5p) further improving discriminatory power (AUC 0.870).

Future studies are needed in larger patient cohorts to validate the application of these identified plasma EV miRNAs for lung disease differentiation and prediction, as well as explore their potential mechanisms in lung disease progression. 

## Figures and Tables

**Table 1:** Clinical characteristics of healthy non-smokers (n = 20), healthy smokers (n = 20), lung cancer (n = 20) and COPD (stable, n = 20) participants.

Clinical Characteristic	Healthy Non-Smokers	Healthy Smokers	Lung Cancer	COPD (Stable)	Total	Significance
Gender (n, %)						
Males	7 (35)	13 (65)	10 (50)	12 (60)	42 (52.5)	*p* = 0.240 " ^1^
Females	13 (65)	7 (35)	10 (50)	8 (40)	38 (47.5)	
Age (mean, SD)	55.1 (7.0)	60.4 (4.2)	66.7 (7.3)	70.3 (7.6)	63.2 (8.6)	*p* < 0.0001 * " ^2^
Smoking history (n, %)						
Never	20 (100)	0	0	1 (5)	21 (26.3)	*p* = 0.02 * " ^2^
Former	-	8 (40)	14 (70)	15 (75)	37 (46.3)	
Current	-	12 (60)	5 (25)	4 (20)	22 (27.5)	
Pack years (mean, SD)	-	46.2 (15.8)	39.8 (23.2)	58.2 (23.2)	48.3 (21.8)	*p* = 0.058 " ^2^
TNM (n, %)						
IA or B	-	-	9 (45)	-	-	-
IIA or B	-	-	2 (10)	-	-	-
IIIA or B	-	-	1 (5)	-	-	-
IVA or B	-	-	6 (30)	-	-	-
NSCLC subtype						
Adenocarcinoma			14 (70)			
Squamous cell carcinoma			2 (10)			
Non-small cell carcinoma			4 (20)			
GOLD stage (n, %)						
GOLD 0	-	-	-	3 (15)	-	-
GOLD 1	-	-	-	0	-	-
GOLD 2	-	-	-	8 (40)	-	-
GOLD 3	-	-	-	5 (25)	-	-
GOLD 4	-	-	-	4 (20)	-	-

^1^ Categorical data represented as n (population %); continuous data represented as mean (standard deviation); * *p*-value < 0.05; " non-normal distribution; ^1^Chi-square test; ^2^ Kruskal–Wallis test.

**Table 2:** Over-and under-expressed miRNA targets that were identified to be significantly (*p* < 0.05) dysregulated between lung cancer participants and comparator cohorts (healthy non-smokers, healthy smokers and stable COPD participants).

	Healthy Non-Smokers	Healthy Smokers	COPD (Stable)
**Lung cancer**	**miRNAs underexpressed (fold regulation; significance)**		
	hsa-miR-221-3p (-2.2; *p* = 0.001 ^1^) hsa-miR-200a-3p (-3.43; *p* = 0.013 ^1^) hsa-miR-133b (-9.08; *p* < 0.0001 ^1^) hsa-miR-497-5p (-4.76; *p* = 0.001 ^2^) hsa-miR-136-5p (-3.6; *p* = 0.01 ^2^) hsa-miR-326 (-4.47; *p* = 0.011 ^2^) hsa-miR-107 (-2.27; *p* < 0.0001 ^1^) hsa-miR-205-5p (-4.45; *p* = 0.0001 ^2^) hsa-miR-339-5p (-2.62; *p* = 0.002 ^1^) hsa-miR-122-5p (-2.6; *p* = 0.032 ^2^) hsa-miR-103a-3p (-2.14; *p* < 0.0001 ^2^) hsa-miR-210-3p (-2.37; *p* = 0.0002 ^2^) hsa-miR-199a-5p (-8.17; *p* = 0.0005 ^2^)	hsa-miR-133b (-6.69; *p* = 0.0001 ^1^) hsa-miR-133a-3p (-4.9; *p* = 0.017 ^2^) hsa-miR-451a (-2.18; *p* < 0.0001 ^2^) hsa-miR-497-5p (-5.12; *p* = 0.0001 ^2^) hsa-miR-107 (-2.19; *p* < 0.0001 ^1^) hsa-miR-205-5p (-3.74; *p* = 0.003 ^2^) hsa-miR-22-5p (-2.07; *p* = 0.002 ^2^) hsa-miR-103a-3p (-2.02; *p* < 0.0001 ^1^) hsa-miR-210-3p (-2.14; *p* = 0.001 ^2^) hsa-miR-199a-5p (-4.56; *p* = 0.009 ^2^) hsa-miR-144-3p (-2.17; *p* < 0.0001 ^1^) hsa-miR-7-5p (-2.85; *p* = 0.014 ^2^)	hsa-miR-133b (-4.75; *p* = 0.001 ^2^) hsa-miR-130a-3p (-2.09; *p* < 0.0001 ^1^) hsa-miR-497-5p (-3.15; *p* = 0.008 ^2^) hsa-miR-1260a (-2.53; *p* < 0.0001 ^1^) hsa-miR-210-3p (-2.29; *p* = 0.0003 ^2^) hsa-miR-543 (-2.36; *p* = 0.027 ^2^)
	**miRNAs overexpressed (fold regulation; significance)**		
	hsa-miR-197-3p (2.45; *p* = 0.019 ^2^) hsa-miR-223-3p (2.49; *p* = 0.017 ^2^)	hsa-miR-505-3p (2.27; *p* = 0.022 ^2^) hsa-miR-23b-3p (2.12; *p* = 0.002 ^2^) hsa-miR-361-5p (2.18; *p* = 0.001 ^2^) hsa-miR-27a-3p (2.06; *p* = 0.0002 ^1^) hsa-miR-328-3p (2.55; *p* = 0.007 ^2^) hsa-let-7b-3p (2.05; *p* = 0.002 ^2^) hsa-miR-425-3p (2.12; *p* = 0.021 ^2^) hsa-miR-197-3p (3.31; *p* < 0.0001 ^1^) hsa-miR-223-5p (3.75; *p* = 0.002 ^2^) hsa-miR-191-5p (2.11; *p* < 0.0001 ^1^) hsa-miR-23a-3p (2.01; *p* = 0.007 ^2^) hsa-miR-423-3p (2.26; *p* = 0.007 ^1^) hsa-miR-223-3p (4.52; *p* < 0.0001 ^2^) hsa-miR-24-3p (2.12; *p* = 0.0002 ^1^)	hsa-miR-361-5p (2.08; *p* = 0.002 ^1^) hsa-miR-27a-3p (2.06; *p* = 0.0005 ^1^) hsa-miR-197-3p (2.26; *p* = 0.004 ^1^) hsa-miR-223-5p (3; *p* = 0.042 ^2^) hsa-miR-193a-5p (2.18; *p* = 0.007 ^2^) hsa-miR-423-3p (2.23; *p* = 0.01 ^1^) hsa-miR-223-3p (3.54; *p* = 0.001 ^2^) hsa-miR-106b-3p (3.37; *p* = 0.004 ^2^)

^1^ Independent t-test; ^2^ Mann–Whitney U test.

**Table 3:** Predicted biological pathways enriched by the significantly dysregulated miRNAs as identified in the primary analyses of lung cancer participants compared to healthy non-smoking participants.

Patient Groups	KEGG Pathway	Significance	# miRNAs	List of Identified Target Genes	# Genes Validated from GeneGlobe Analysis
Lung cancer vs. healthy non-smokers	Fatty acid biosynthesis (hsa00061)	*p* < 0.0001 ^1^	2	FASN ACSL3	2
	Fatty acid metabolism (hsa01212)	*p* < 0.0001 ^1^	10	FASN ACSL3 PTPLB SCD5 ACAT2 ELOVL2 ELOVL6 ACAT1 ACADM MECR	4
	Proteoglycans in cancer (hsa05205)	*p* = 0.009 ^1^	12	BRAF WNT16 WNT7A SOS2 CBL ROCK2 FZD6 ITGA5 MRAS ANK2 COL21A1 ANK3 SLC9A1 FZD4 FZD10 IGF2 EIF4B DDX5 PLAUR FGF2 TGFB2 ANK1 AKT3 WNT3A SMO VEGFA FGFR1 KDR GRB2 WNT7B MDM2 ELK1 PRKACB	21

^1^ Gene union criterion.

## Data Availability

Data is contained within the article or supplementary material.

## Supplementary Materials

The following are available online at https://www.mdpi.com/article/10.3390/ijms22115803/s1, Table S1: The top miRNA-regulated genes targeting miRNAs that were identified as significantly over- or under-expressed in lung disease groups (healthy smokers, lung cancer & stable COPD) compared to healthy controls and between different lung disease groups, Table S2: Correlation analysis between clinical characteristics and identified significantly dysregulated miRNAs for lung cancer participants compared to healthy non-smokers, Table S3: Significantly associated miRNAs with categorical clinical characteristics, including gender and smoking history Table S4: Correlation analysis between clinical characteristics and identified significantly dysregulated miRNAs for lung cancer participants compared to healthy smokers, Table S5: Correlation analysis between clinical characteristics and identified significantly dysregulated miRNAs for lung cancer participants compared to stable COPD participants.
