# Supplementary material for: Plasma Extracellular Vesicle miRNAs Can Identify Lung Cancer, Current Smoking Status, and Stable COPD
Source: Int J Mol Sci. 2021 May 28;22(11):5803. doi: 10.3390/ijms22115803 (PMC8198071; doi:10.3390/ijms22115803)
Supplement: Supplementary file 1 [file ijms-22-05803-s001.zip › ijms-1223750-supplementary.pdf]

Supplementary

Plasma extracellular vesicle miRNAs can identify lung cancer, current smoking status and stable COPD

Hannah E. O'Farrell[1], Rayleen V. Bowman[1], Kwun M. Fong[1], Ian A. Yang[1].

[1] The University of Queensland Thoracic Research Centre, The Prince Charles Hospital, Brisbane, QLD, Australia

Corresponding author

Hannah E. O'Farrell

UQ Thoracic Research Centre

Level 1, Clinical Sciences Building, The Prince Charles Hospital

Rode Road, Chermside, QLD, 4032

Australia

Phone: +61 07 3139 4110

Email: hannah.ofarrell@uq.net.au

Methods

Blood plasma sample collection and processing

Peripheral blood was obtained from 80 patients (20 healthy controls, 20 healthy smokers, 20 patients with a diagnosis of lung cancer, and 20 patients with stable chronic obstructive pulmonary disease) and processed to separate the plasma from the blood cell fraction. Briefly peripheral blood was drawn into x2-5 BD vacutainer K2 EDTA plus blood collection tubes (maximum volume for x1 tube = 4mL). Blood fractionation was performed by differential centrifugation, firstly by centrifuging the whole blood at 1200xg for 10 min at room temperature. This was followed by the plasma supernatant then being centrifuged at 1600xg for 10 min at room temperature in order to remove apoptotic bodies, cellular fragments and circulating tumour cells [1]. The plasma supernatant was then centrifuged at 20,000xg for 40min at 4˚C in order to deplete larger microvesicles. The remaining larger microvesicle-depleted plasma supernatant was then bio-banked and stored at -80˚C until further downstream EV isolation.

Characterisation of blood plasma extracellular vesicles

Nanoparticle tracking analysis

Re-suspended EVs in 1X PBS were analysed by nanoparticle tracking analysis (NTA) using the NanoSight NS300 instrument (Malvern Instruments, Amesbury, UK). This model is equipped with a blue 488nm laser, a high sensitivity SCMOS camera and syringe pump. The samples were further filtered with 1X PBS using a 0.4 μm Minisart filter (Sartorius, Germany) to obtain 40–100 particles/view. Samples were then loaded into a 1mL syringe and connected to the syringe pump and run at a speed of 50, with an automatic temperature setting of 22-24°C. Ten 60s videos were then recorded using camera level 1.8-2.2 and a screen gain of 10-11. Data was analysed using NTA software (v3.2) with the detection threshold set at 4-5 and screen gain at 10-11 in order to track the most particles with minimal background. The Brownian motion of particles is used to determine EV particle size through dynamic light scattering [2].

Western blot of EV markers

Protein concentration of re-suspended EVs in 1X PBS was assessed using the Pierce BCA Protein Assay Kit (ThermoFisher Scientific, USA) as per the manufacturer's instructions. Reconstituted lyophilized exosome standard from human plasma (HansaBiomed, Estonia) was used as a positive control. 5-10ug of re-suspended EVs were then prepared in Bolt LDS sample buffer (ThermoFisher Scientific, USA) under reducing conditions and boiled for 5 mins at 95˚C, followed by 10 minutes on ice. Proteins were separated by SDS-PAGE (SDS-polyacrylamide gel electrophoresis) and transferred onto polyvinylidene difluoride (PVDF) membranes, blocked in 5% non-fat powdered milk in Tris buffered

saline and 0.5%Tween-20 (TBS-T) and then probed with antibodies. Primary antibodies included human Albumin, Flotillin-1, diluted 1:1000 (Cell Signalling Technology, 4929, 18634 respectively) and anti-CD9, diluted 1:2000 (Abcam, EPR2949). Proteins of interest were detected with 1:10,000 diluted Goat anti-Rabbit HRP-conjugated IgG (H+L) antibody in TBS-T (Invitrogen, USA) and visualized with SuperSignal West Pico PLUS Chemiluminescent Substrate (ThermoFisher Scientific, USA).

Isolation and RNA extraction of blood plasma extracellular vesicles

EV isolation

Frozen plasma samples were thawed and isolated for EVs using the commercially available miRCURY Exosome Serum/Plasma Kit (Qiagen, Hilden, Germany) according to the manufacturer's instructions. Briefly, 600μL of thawed plasma was incubated with thrombin for defibrination, followed by EV precipitation with 500μL of the thrombin treated plasma and 0.4 volumes of Precipitation Buffer A. Samples were then incubated at 4˚C for 1 hour and then centrifuged at 500xg for 5 min at 20˚C. The supernatant was then separated and the remaining EV pellet was either re-suspended in 200μL 1X PBS, and stored at -80 for downstream characterisation, or re-suspended in 700μL QIAZOL as per the manufacturers protocol for RNA purification using the miRNeasy Mini Kit (QIAGEN, Hilden, Germany).

EV RNA purification

Total RNA was extracted using the miRNeasy Mini Kit (Qiagen, Hilden, Germany) from the 700μL QIAZOL re-suspended pelleted EVs from the initial 500μL of thrombin pre-treated plasma. To improve RNA yield and reproducibility between samples, 1.25μg/mL of MS2 bacteriophage carrier RNA (Roche Diagnostics GmbH, Mannheim, Germany) was added along with the recommended synthetic RNA spike-ins (UniSp2, UniSp4, UniSp5 – control for RNA isolation efficiency, as per the manufactures protocol, Qiagen, Hilden, Germany) during the QIAZOL re-suspension of the EV pellet. Total RNA was extracted using the QIACube and eluted with 50μL of ribonuclease-free water, then stored at -80˚C prior to miRNA profiling.

Blood plasma extracellular vesicle miRNA profiling

Reverse transcription

Reverse transcription (RT) of total RNA was performed with the miRCURY LNA RT kit (QIAGEN, Hilden, Germany) as per the manufacturer's instructions, using UniSp6 and cel-39 spike-ins as internal controls to detect the presence of potential inhibitors. As the amount of RNA in plasma cannot be accurately determined, it was recommended as per the manufacturer's protocols to calculate the

volume of RNA input corresponding to 16µL of original plasma sample for each 20µL RT reaction (e.g. for RNA isolated from 500µL of plasma and eluted in 50µL, use 1.6µL of eluate in each RT reaction).

Quantitative PCR using miRCURY LNA Serum/Plasma Focus PCR Panels

Samples from the RT reaction were prepared with the miRCURY SYBR Green PCR Kit (QIAGEN, Hilden, Germany) and assessed for miRNA gene expression using the miRCURY LNA miRNA Serum/Plasma Focus PCR Panels (QIAGEN, Hilden, Germany) in a 384 well format, which detects 179 of the most expressed miRNAs in human plasma. Amplification was performed using the ViiA 7 real time PCR system (Applied Biosystems) with a low concentration of ROX reference dye (20X concentration) as per the manufacturer's protocol. Results reported as Ct values were obtained using the QuantStudio v6 software, with the raw Ct values being exported into an Excel worksheet and uploaded onto the QIAGEN data analysis web portal at http://www.giagen.com/geneglobe.

GeneGlobe was utilised for analysis of internal amplification controls, calibration of raw Ct data for plate-to-plate variations using the inter-plate calibrator controls (UniSp3 IPC), haemolysis testing and setting Ct cut-off values, which only miRNAs with a Ct <40 were considered for analysis. Haemolysis is indicated with a ΔCt difference between miR-23a and miR-451 >7. Samples were still included with ΔCt difference <9 as it has been reported that while haemolysed blood products contain higher concentrations of certain RNAs (including miR-451) [3], it is still undetermined if these RNAs confound results in EV RNA analysis [4].

Samples were assigned to a control group (healthy participants) or test groups (group 1 = healthy smokers; group 2 = lung cancer; group 3 = stable COPD). Processed Ct values were normalised based on the NormFinder algorithm supplied by the GeneGlobe software [5-7]. NormFinder ranks a set of candidate normalisation miRNAs according to their expression stability. Optimum reference miRNAs identified and selected included miR-140-3p, miR-222-3p, miR-15a-5p, miR-25-3p, miR-152-3p, miR-30e-5p, miR-19a-3p, let-7g-5p, miR-19b-3p and miR-21-5p.

Fold change/ regulation were calculated using the web portal and the ΔΔCt method, where the ΔCt is calculated between the miRNA of interest and the arithmetic mean average of the selected reference miRNAs, followed by ΔΔCt calculations (ΔCt test group – ΔCt control group). Fold change was then calculated using 2^ (-ΔΔCt) formula.

miRNA Function Enrichment Analysis

Kyoto Encyclopaedia of Genes and Genomes (KEGG) pathway analysis was performed using the online software DIANA-mirPath v.3.0, with identified miRNA targets searched also using TargetScan [8]. Results were merged by a "gene union" or "pathway union" criterion with significance calculated by the DIANA software using p-value threshold <0.05, TargetScan Score Type of Context + (-0.4) or a TargetScan Conservation Score (0.1) and a Fisher's Exact Test being the statistical test for the enrichment analysis.

Statistical analyses

All statistical analyses was performed by using GeneGlobe (http://www.giagen.com/geneglobe), DIANA-miRPath v.3.0 [8] and Statistical Package for Social Science (SPSS v.27.0) (IBM, NY, USA). A p-value of <0.05 (two-tailed) was statistically significant.

Relative miRNA expression was assessed for normality using the Shapiro-Wilk test. The significance of the identified miRNAs with a fold regulation/ fold change >2 was assessed using either an independent t-test (normally distributed data) or Mann-Whitney U test (non-normally distributed data). Fold-change values greater than one indicates an over-expression, and the fold-regulation is equal to the fold-change. Fold-change values less than one indicate an under-expression, and the fold-regulation is the negative inverse of the fold-change.

The differences in clinical characteristics between patient cohorts was analysed by Chi square test for categorical variables or depending on normal distribution of the data, an independent t-test (normal distribution) or Mann-Whitney U test (non-normal distribution) for continuous variables. Spearman Rank correlation analysis was performed between continuous clinical characteristics and miRNA relative expression (ΔCt values).

For the significantly dysregulated miRNAs identified in plasma EVs, receiver operating characteristic (ROC) curves and area under the curve (AUC) values with 95% confidence intervals (CIs) were calculated to determine the diagnostic potential these miRNAs have in discriminating between test and control groups. A multivariate logistic regression model was established with combinations of suitable miRNAs and appropriate clinical characteristics, which was then used to evaluate the association of differentially expressed miRNAs and the risk of lung disease. If miRNA combinations included both over- and under-expressed targets, under-expressed miRNAs ΔCt values were multiplied with -1 for input into the logistic regression model analysis.

Supplementary Tables

Table S1. The top miRNA-regulated genes targeting miRNAs that were identified as significantly over- or under-expressed in lung disease groups (healthy smokers, lung cancer & stable COPD) compared to healthy controls and between different lung disease groups.

Patient Groups	Gene	# of miRNA Targeting this Gene	Total # of Target Sites Identified in Target Gene	Range of Strength Scores (min to max)	miRNAs Targeting this Gene
Lung cancer vs. Healthy controls	**Under-expressed**				
	DICER1	4	9	-0.397 - -0.033	hsa-miR-107,hsa-miR-497-5p,hsa-miR-221-3p,hsa-miR-122-5p
	**Over-expressed**				
	FBXW7	2	4	-0.463 - -0.313	hsa-miR-223-3p,hsa-miR-197-3p
	ZXDC	2	3	-0.309 - -0.141	hsa-miR-223-3p,hsa-miR-197-3p
	HLF	1	3	-0.161 - -0.0652	hsa-miR-223-3p
	ARMC1	2	2	-0.43 - -0.396	hsa-miR-197-3p,hsa-miR-223-3p
	BRWD1	2	2	-0.1833 - -0.1332	hsa-miR-197-3p,hsa-miR-223-3p
	EBF3	2	2	-0.196	hsa-miR-197-3p,hsa-miR-223-3p
	HIPK2	2	2	-0.177	hsa-miR-223-3p,hsa-miR-197-3p
	SOX11	2	2	-0.038	hsa-miR-223-3p,hsa-miR-197-3p
	UBE2W	2	2	-0.127	hsa-miR-223-3p,hsa-miR-197-3p
	VTI1A	2	2	-0.063	hsa-miR-197-3p,hsa-miR-223-3p
Lung cancer	**Under-expressed**				

vs.<br>Healthy smokers	*ZNF148*	4	8	-0.218 - -0.037	hsa-miR-144-3p,hsa-miR-7-5p,hsa-miR-205-5p,hsa-miR-199a-5p
	DICER1	3	8	-0.397 - -0.033	hsa-miR-107,hsa-miR-144-3p,hsa-miR-497-5p
	LDLRAP1	2	8	-0.315 - -0.006	hsa-miR-133a-3p,hsa-miR-133b
	MECP2	5	7	-0.13 - -0.051	hsa-miR-107,hsa-miR-7-5p,hsa-miR-205-5p,hsa-miR-133a-3p,hsa-miR-133b
	ZHX3	5	7	-0.35 - -0.082	hsa-miR-205-5p,hsa-miR-133a-3p,hsa-miR-133b,hsa-miR-107,hsa-miR-497-5p
	**Over-expressed**				
	NFIB	5	11	-0.366 - 0.007	hsa-miR-223-3p,hsa-miR-27a-3p,hsa-miR-23a-3p,hsa-miR-23b-3p,hsa-miR-361-5
	ZBTB34	3	10	-0.368 - -0.113	hsa-miR-23b-3p,hsa-miR-23a-3p,hsa-miR-27a-3p
	ETNK1	3	9	-0.365 - -0.151	hsa-miR-23a-3p,hsa-miR-23b-3p,hsa-miR-24-3p
	ZFHX4	3	9	-0.222 - -0.124	hsa-miR-27a-3p,hsa-miR-23a-3p,hsa-miR-23b-3p

	EBF3	7	8	-0.456 - -0.069	hsa-miR-23a-3p,hsa-miR-23b-3p,hsa-miR-197-3p,hsa-miR-328-3p,hsa-miR-24-3p,hsa-miR-223-3p,hsa-miR-27a-3p
Lung cancer vs. COPD (Stable)	**Over-expressed**				
	FBXW7	3	6	-0.473 - -0.313	hsa-miR-223-3p,hsa-miR-27a-3p,hsa-miR-197-3p
	EBF3	4	5	-0.289 - -0.069	hsa-miR-197-3p,hsa-miR-223-3p,hsa-miR-27a-3p,hsa-miR-193a-5p
	HIPK2	3	5	-0.397 - -0.124	hsa-miR-27a-3p,hsa-miR-223-3p,hsa-miR-197-3p
	NFIB	3	5	-0.366 - 0.007	hsa-miR-223-3p,hsa-miR-27a-3p,hsa-miR-361-5p
	SOX11	3	4	-0.382 - -0.136	hsa-miR-27a-3p,hsa-miR-223-3p,hsa-miR-197-3p
	**Under-expressed**				
	APLN	1	7	-0.13 - 0.02	hsa-miR-497-5p
	CLCN5	4	6	-0.3723 - -0.0791	hsa-miR-130a-3p,hsa-miR-497-5p,hsa-miR-543,hsa-miR-200a-3p
	FBXO21	2	5	-0.299- -0.055	hsa-miR-497-5p, hsa-miR-543

	IPPK	3	4	-0.249 - -0.021	hsa-miR-543, hsa-miR-497-5p, ,hsa-miR-1260a
	LRP6	3	4	-0.289 - -0.045	hsa-miR-497-5p,hsa-miR-543, hsa-miR-130a-3p

Table S2. Correlation analysis between clinical characteristics and identified significantly dysregulated miRNAs for lung cancer participants compared to healthy non-smokers.

		Age	hsa-miR-197-3p	hsa-miR-223-3p	hsa-miR-221-3p	hsa-miR-200a-3p	hsa-miR-133b	hsa-miR-497-5p	hsa-miR-136-5p	hsa-miR-326	hsa-miR-107	hsa-miR-205-5p	hsa-miR-339-5p	hsa-miR-122-5p	hsa-miR-103a-3p	hsa-miR-210-3p	hsa-miR-199a-5p
**Age**	Correlation Coefficient	1.000	-0.284	-0.148	0.263	0.144	0.217	0.093	0.085	0.173	.537**	0.263	0.221	-0.023	.556**	0.235	0.243
	Sig. (2-tailed)		0.075	0.363	0.102	0.374	0.178	0.570	0.602	0.286	0.000	0.101	0.170	0.887	0.000	0.145	0.131
	N	40	40	40	40	40	40	40	40	40	40	40	40	40	40	40	40
**hsa-miR-197-3p**	Correlation Coefficient	-0.284	1.000	.743**	0.294	-0.092	-.399*	-0.229	0.015	-0.072	-.503**	-0.258	-0.076	-0.292	-.479**	-.460**	-0.250
	Sig. (2-tailed)	0.075		0.000	0.066	0.574	0.011	0.156	0.925	0.658	0.001	0.107	0.640	0.067	0.002	0.003	0.119
	N	40	40	40	40	40	40	40	40	40	40	40	40	40	40	40	40
**hsa-miR-223-3p**	Correlation Coefficient	-0.148	.743**	1.000	0.258	-0.207	-.405**	-0.310	0.036	0.000	-.456**	-.366*	0.038	-.459**	-.411**	-.440**	-0.131
	Sig. (2-tailed)	0.363	0.000		0.109	0.200	0.010	0.052	0.824	0.999	0.003	0.020	0.816	0.003	0.008	0.004	0.419
	N	40	40	40	40	40	40	40	40	40	40	40	40	40	40	40	40
**hsa-miR-221-3p**	Correlation Coefficient	0.263	0.294	0.258	1.000	.337*	.326*	.345*	.331*	.419**	0.258	0.225	0.294	-0.005	.353*	0.298	.410**
	Sig. (2-tailed)	0.102	0.066	0.109		0.033	0.040	0.029	0.037	0.007	0.108	0.163	0.066	0.978	0.026	0.062	0.009

	N	40	40	40	40	40	40	40	40	40	40	40	40	40	40	40	40
**hsa-miR-200a-3p**	Correlation Coefficient	0.144	-0.092	-0.207	.337*	1.000	0.143	0.238	0.048	0.020	.405**	.431**	0.255	.371*	.371*	.325*	0.201
	Sig. (2-tailed)	0.374	0.574	0.200	0.033		0.379	0.140	0.768	0.900	0.010	0.005	0.112	0.018	0.018	0.041	0.213
	N	40	40	40	40	40	40	40	40	40	40	40	40	40	40	40	40
**hsa-miR-133b**	Correlation Coefficient	0.217	-.399*	-.405**	.326*	0.143	1.000	.458**	0.312	.370*	.588**	.375*	0.104	0.302	.625**	.657**	.466**
	Sig. (2-tailed)	0.178	0.011	0.010	0.040	0.379		0.003	0.050	0.019	0.000	0.017	0.524	0.058	0.000	0.000	0.002
	N	40	40	40	40	40	40	40	40	40	40	40	40	40	40	40	40
**hsa-miR-497-5p**	Correlation Coefficient	0.093	-0.229	-0.310	.345*	0.238	.458**	1.000	.458**	0.273	.490**	.627**	0.192	.583**	.455**	.466**	.521**
	Sig. (2-tailed)	0.570	0.156	0.052	0.029	0.140	0.003		0.003	0.088	0.001	0.000	0.236	0.000	0.003	0.002	0.001
	N	40	40	40	40	40	40	40	40	40	40	40	40	40	40	40	40
**hsa-miR-136-5p**	Correlation Coefficient	0.085	0.015	0.036	.331*	0.048	0.312	.458**	1.000	.499**	0.281	0.242	.426**	0.093	0.228	0.093	.526**
	Sig. (2-tailed)	0.602	0.925	0.824	0.037	0.768	0.050	0.003		0.001	0.079	0.132	0.006	0.567	0.157	0.570	0.000
	N	40	40	40	40	40	40	40	40	40	40	40	40	40	40	40	40
**hsa-miR-326**	Correlation Coefficient	0.173	-0.072	0.000	.419**	0.020	.370*	0.273	.499**	1.000	0.297	0.095	0.204	0.250	.361*	0.306	.392*
	Sig. (2-tailed)	0.286	0.658	0.999	0.007	0.900	0.019	0.088	0.001		0.063	0.559	0.207	0.120	0.022	0.055	0.012
	N	40	40	40	40	40	40	40	40	40	40	40	40	40	40	40	40

**hsa-miR-107**	Correlation Coefficient	.537**	-.503**	-.456**	0.258	.405**	.588**	.490**	0.281	0.297	1.000	.611**	.340*	.338*	.923**	.573**	.455**
	Sig. (2-tailed)	0.000	0.001	0.003	0.108	0.010	0.000	0.001	0.079	0.063		0.000	0.032	0.033	0.000	0.000	0.003
	N	40	40	40	40	40	40	40	40	40	40	40	40	40	40	40	40
**hsa-miR-205-5p**	Correlation Coefficient	0.263	-0.258	-.366*	0.225	.431**	.375*	.627**	0.242	0.095	.611**	1.000	0.163	.538**	.544**	.538**	0.233
	Sig. (2-tailed)	0.101	0.107	0.020	0.163	0.005	0.017	0.000	0.132	0.559	0.000		0.315	0.000	0.000	0.000	0.148
	N	40	40	40	40	40	40	40	40	40	40	40	40	40	40	40	40
**hsa-miR-339-5p**	Correlation Coefficient	0.221	-0.076	0.038	0.294	0.255	0.104	0.192	.426**	0.204	.340*	0.163	1.000	0.017	.333*	0.101	0.295
	Sig. (2-tailed)	0.170	0.640	0.816	0.066	0.112	0.524	0.236	0.006	0.207	0.032	0.315		0.918	0.036	0.534	0.064
	N	40	40	40	40	40	40	40	40	40	40	40	40	40	40	40	40
**hsa-miR-122-5p**	Correlation Coefficient	-0.023	-0.292	-.459**	-0.005	.371*	0.302	.583**	0.093	0.250	.338*	.538**	0.017	1.000	.336*	.477**	0.212
	Sig. (2-tailed)	0.887	0.067	0.003	0.978	0.018	0.058	0.000	0.567	0.120	0.033	0.000	0.918		0.034	0.002	0.188
	N	40	40	40	40	40	40	40	40	40	40	40	40	40	40	40	40
**hsa-miR-103a-3p**	Correlation Coefficient	.556**	-.479**	-.411**	.353*	.371*	.625**	.455**	0.228	.361*	.923**	.544**	.333*	.336*	1.000	.636**	.522**
	Sig. (2-tailed)	0.000	0.002	0.008	0.026	0.018	0.000	0.003	0.157	0.022	0.000	0.000	0.036	0.034		0.000	0.001
	N	40	40	40	40	40	40	40	40	40	40	40	40	40	40	40	40

**hsa-miR-210-3p**	Correlation Coefficient	0.235	-.460**	-.440**	0.298	.325*	.657**	.466**	0.093	0.306	.573**	.538**	0.101	.477**	.636**	1.000	.316*
	Sig. (2-tailed)	0.145	0.003	0.004	0.062	0.041	0.000	0.002	0.570	0.055	0.000	0.000	0.534	0.002	0.000		0.047
	N	40	40	40	40	40	40	40	40	40	40	40	40	40	40	40	40
**hsa-miR-199a-5p**	Correlation Coefficient	0.243	-0.250	-0.131	.410**	0.201	.466**	.521**	.526**	.392*	.455**	0.233	0.295	0.212	.522**	.316*	1.000
	Sig. (2-tailed)	0.131	0.119	0.419	0.009	0.213	0.002	0.001	0.000	0.012	0.003	0.148	0.064	0.188	0.001	0.047	
	N	40	40	40	40	40	40	40	40	40	40	40	40	40	40	40	40

Spearman rank correlation
*P<0.05

Table S3. Significantly associated miRNAs with categorical clinical characteristics, including gender and smoking history.

	Healthy non-smokers	Healthy smokers	Stable COPD
**Lung cancer**	Significantly increased in males		
	miR-223-3p (p=0.05)<br>miR-339-5p (p=0.04)	miR-505-3p (p=0.022)<br>miR-328-3p (p=0.028)<br>miR-197-3p (p=0.03)<br>miR-423-3p (p=0.01)<br>miR-223-3p (p=0.034)	miR-223-3p (p=0.016)
	Significantly increased in females		
	miR-200a-3p (p=0.036)<br>miR-122-5p (p=0.046)	miR-103a-3p (p=0.01)<br>miR-210-3p (p=0.047)	miR-133b (p=0.009)<br>miR-210-3p (p=0.001)
	Significantly increased in former smokers compared to current smokers		
	-	miR-107<br>(p=0.003)<br>miR-103a-3p (p=0.007)<br>miR-144-3p (p=0.012)	NIL

Mann-Whitney U test

Table S4. Correlation analysis between clinical characteristics and identified significantly dysregulated miRNAs for lung cancer participants compared to healthy smokers.

		Age	Pack years	hsa-miR-505-3p	hsa-miR-23b-3p	hsa-miR-361-5p	hsa-miR-27a-3p	hsa-miR-328-3p	hsa-let-7b-3p	hsa-miR-425-3p	hsa-miR-197-3p	hsa-miR-223-5p	hsa-miR-191-5p	hsa-miR-23a-3p	hsa-miR-423-3p
**Age**	Correlation Coefficient	1	0.095	-0.307	-.366*	-.331*	-.323*	-0.095	-.432**	-0.05	-0.261	-0.231	-.458**	-0.284	0.009
	Sig. (2-tailed)		0.575	0.054	0.02	0.037	0.042	0.559	0.005	0.758	0.103	0.152	0.003	0.076	0.954
	N	40	37	40	40	40	40	40	40	40	40	40	40	40	40
**Pack years**	Correlation Coefficient	0.095	1	0.155	-0.073	-0.063	0.139	0.147	-0.079	-0.138	-0.046	0.076	0.095	-0.008	0.061
	Sig. (2-tailed)	0.575		0.358	0.669	0.711	0.411	0.385	0.64	0.417	0.789	0.657	0.577	0.963	0.722
	N	37	37	37	37	37	37	37	37	37	37	37	37	37	37
**hsa-miR-505-3p**	Correlation Coefficient	-0.307	0.155	1	.601**	.550**	.581**	.483**	0.212	.529**	.648**	.575**	.381*	.638**	0.301
	Sig. (2-tailed)	0.054	0.358		0	0	0	0.002	0.189	0	0	0	0.015	0	0.059
	N	40	37	40	40	40	40	40	40	40	40	40	40	40	40
**hsa-miR-23b-3p**	Correlation Coefficient	-.366*	-0.073	.601**	1	.721**	.777**	.538**	.351*	.536**	.757**	.695**	.547**	.871**	.436**
	Sig. (2-tailed)	0.02	0.669	0		0	0	0	0.027	0	0	0	0	0	0.005
	N	40	37	40	40	40	40	40	40	40	40	40	40	40	40
**hsa-miR-361-5p**	Correlation Coefficient	-.331*	-0.063	.550**	.721**	1	.754**	.422**	.341*	.565**	.734**	.559**	.497**	.787**	.407**
	Sig. (2-tailed)	0.037	0.711	0	0		0	0.007	0.031	0	0	0	0.001	0	0.009
	N	40	37	40	40	40	40	40	40	40	40	40	40	40	40
	Correlation Coefficient	-.323*	0.139	.581**	.777**	.754**	1	.484**	.400*	.432**	.682**	.611**	.646**	.872**	.427**

**hsa-miR-27a-3p**	Sig. (2-tailed)	0.042	0.411	0	0	0		0.002	0.01	0.005	0	0	0	0	0.006
	N	40	37	40	40	40	40	40	40	40	40	40	40	40	40
**hsa-miR-328-3p**	Correlation Coefficient	-0.095	0.147	.483**	.538**	.422**	.484**	1	0.14	.522**	.514**	.411**	.353*	.453**	.418**
	Sig. (2-tailed)	0.559	0.385	0.002	0	0.007	0.002		0.388	0.001	0.001	0.008	0.026	0.003	0.007
	N	40	37	40	40	40	40	40	40	40	40	40	40	40	40
**hsa-let-7b-3p**	Correlation Coefficient	-.432**	-0.079	0.212	.351*	.341*	.400*	0.14	1	0.252	.315*	0.273	.515**	.431**	0.027
	Sig. (2-tailed)	0.005	0.64	0.189	0.027	0.031	0.01	0.388		0.116	0.048	0.089	0.001	0.005	0.868
	N	40	37	40	40	40	40	40	40	40	40	40	40	40	40
**hsa-miR-425-3p**	Correlation Coefficient	-0.05	-0.138	.529**	.536**	.565**	.432**	.522**	0.252	1	.680**	.533**	.394*	.491**	.329*
	Sig. (2-tailed)	0.758	0.417	0	0	0	0.005	0.001	0.116		0	0	0.012	0.001	0.038
	N	40	37	40	40	40	40	40	40	40	40	40	40	40	40
**hsa-miR-197-3p**	Correlation Coefficient	-0.261	-0.046	.648**	.757**	.734**	.682**	.514**	.315*	.680**	1	.598**	.646**	.728**	.528**
	Sig. (2-tailed)	0.103	0.789	0	0	0	0	0.001	0.048	0		0	0	0	0
	N	40	37	40	40	40	40	40	40	40	40	40	40	40	40
**hsa-miR-223-5p**	Correlation Coefficient	-0.231	0.076	.575**	.695**	.559**	.611**	.411**	0.273	.533**	.598**	1	.586**	.667**	.514**
	Sig. (2-tailed)	0.152	0.657	0	0	0	0	0.008	0.089	0	0		0	0	0.001
	N	40	37	40	40	40	40	40	40	40	40	40	40	40	40
**hsa-miR-191-5p**	Correlation Coefficient	-.458**	0.095	.381*	.547**	.497**	.646**	.353*	.515**	.394*	.646**	.586**	1	.540**	.427**
	Sig. (2-tailed)	0.003	0.577	0.015	0	0.001	0	0.026	0.001	0.012	0	0		0	0.006
	N	40	37	40	40	40	40	40	40	40	40	40	40	40	40
	Correlation Coefficient	-0.284	-0.008	.638**	.871**	.787**	.872**	.453**	.431**	.491**	.728**	.667**	.540**	1	.456**

**hsa-miR-23a-3p**	Sig. (2-tailed)	0.076	0.963	0	0	0	0	0.003	0.005	0.001	0	0	0		0.003
	N	40	37	40	40	40	40	40	40	40	40	40	40	40	40
**hsa-miR-423-3p**	Correlation Coefficient	0.009	0.061	0.301	.436**	.407**	.427**	.418**	0.027	.329*	.528**	.514**	.427**	.456**	1
	Sig. (2-tailed)	0.954	0.722	0.059	0.005	0.009	0.006	0.007	0.868	0.038	0	0.001	0.006	0.003	
	N	40	37	40	40	40	40	40	40	40	40	40	40	40	40
**hsa-miR-223-3p**	Correlation Coefficient	-0.171	0.139	.553**	.745**	.646**	.755**	.556**	0.187	.592**	.823**	.688**	.684**	.673**	.612**
	Sig. (2-tailed)	0.291	0.414	0	0	0	0	0	0.249	0	0	0	0	0	0
	N	40	37	40	40	40	40	40	40	40	40	40	40	40	40
**hsa-miR-24-3p**	Correlation Coefficient	-.376*	0.05	.593**	.793**	.816**	.890**	.601**	.496**	.521**	.726**	.547**	.606**	.852**	.404**
	Sig. (2-tailed)	0.017	0.769	0	0	0	0	0	0.001	0.001	0	0	0	0	0.01
	N	40	37	40	40	40	40	40	40	40	40	40	40	40	40
**hsa-miR-133b**	Correlation Coefficient	0.175	-0.18	-.434**	-.330*	-.412**	-.515**	-.361*	-0.151	-0.245	-.469**	-.330*	-.504**	-.321*	-0.26
	Sig. (2-tailed)	0.281	0.285	0.005	0.037	0.008	0.001	0.022	0.353	0.128	0.002	0.037	0.001	0.043	0.105
	N	40	37	40	40	40	40	40	40	40	40	40	40	40	40
**hsa-miR-133a-3p**	Correlation Coefficient	0.176	-0.166	-0.084	-0.143	-0.308	-0.099	-0.147	-0.062	-0.2	-0.268	-.374*	-0.268	-0.08	-0.011
	Sig. (2-tailed)	0.276	0.325	0.608	0.378	0.053	0.545	0.365	0.702	0.215	0.095	0.018	0.094	0.626	0.946
	N	40	37	40	40	40	40	40	40	40	40	40	40	40	40
**hsa-miR-451a**	Correlation Coefficient	.371*	-0.234	-.502**	-.625**	-.610**	-.663**	-.503**	-.496**	-.411**	-.610**	-.531**	-.679**	-.639**	-.421**
	Sig. (2-tailed)	0.019	0.163	0.001	0	0	0	0.001	0.001	0.008	0	0	0	0	0.007
	N	40	37	40	40	40	40	40	40	40	40	40	40	40	40
	Correlation Coefficient	0.206	-0.186	-0.31	-0.113	-0.233	-0.188	-0.305	-.320*	-0.306	-.471**	-0.311	-.569**	-0.08	-.338*

**hsa-miR-497-5p**	Sig. (2-tailed)	0.203	0.269	0.051	0.489	0.147	0.246	0.055	0.044	0.055	0.002	0.051	0	0.624	0.033
	N	40	37	40	40	40	40	40	40	40	40	40	40	40	40
**hsa-miR-107**	Correlation Coefficient	.491**	-0.085	-.508**	-.526**	-.514**	-.565**	-.523**	-.458**	-.391*	-.637**	-.493**	-.692**	-.451**	-.315*
	Sig. (2-tailed)	0.001	0.618	0.001	0	0.001	0	0.001	0.003	0.013	0	0.001	0	0.004	0.048
	N	40	37	40	40	40	40	40	40	40	40	40	40	40	40
**hsa-miR-205-5p**	Correlation Coefficient	0.196	-0.067	-0.233	-0.131	-0.073	-0.136	-.317*	-0.206	-0.3	-0.277	-.374*	-.533**	-0.012	-.407**
	Sig. (2-tailed)	0.225	0.692	0.147	0.421	0.653	0.402	0.046	0.202	0.06	0.084	0.017	0	0.941	0.009
	N	40	37	40	40	40	40	40	40	40	40	40	40	40	40
**hsa-miR-22-5p**	Correlation Coefficient	0.087	-0.119	-0.1	-0.131	-0.276	-.317*	-0.138	-0.182	-0.136	-.316*	-0.251	-.425**	-0.199	-0.217
	Sig. (2-tailed)	0.594	0.483	0.538	0.422	0.085	0.046	0.397	0.262	0.403	0.047	0.119	0.006	0.217	0.178
	N	40	37	40	40	40	40	40	40	40	40	40	40	40	40
**hsa-miR-103a-3p**	Correlation Coefficient	.351*	-0.125	-.498**	-.437**	-.581**	-.590**	-.542**	-.487**	-.465**	-.661**	-.381*	-.684**	-.441**	-0.299
	Sig. (2-tailed)	0.026	0.46	0.001	0.005	0	0	0	0.001	0.002	0	0.015	0	0.004	0.061
	N	40	37	40	40	40	40	40	40	40	40	40	40	40	40
**hsa-miR-210-3p**	Correlation Coefficient	.378*	0.04	-.509**	-.458**	-.494**	-.531**	-.316*	-0.134	-0.306	-.434**	-.571**	-.543**	-.421**	-.360*
	Sig. (2-tailed)	0.016	0.813	0.001	0.003	0.001	0	0.047	0.409	0.055	0.005	0	0	0.007	0.023
	N	40	37	40	40	40	40	40	40	40	40	40	40	40	40
**hsa-miR-199a-5p**	Correlation Coefficient	0.146	0.014	-0.146	-0.234	-.425**	-0.188	-0.191	-0.288	-0.302	-.314*	-.351*	-.493**	-0.26	-0.224
	Sig. (2-tailed)	0.368	0.934	0.37	0.147	0.006	0.246	0.238	0.071	0.058	0.049	0.026	0.001	0.105	0.165
	N	40	37	40	40	40	40	40	40	40	40	40	40	40	40
	Correlation Coefficient	.391*	-0.179	-.566**	-.688**	-.749**	-.734**	-.549**	-.495**	-.591**	-.777**	-.577**	-.763**	-.683**	-.417**

**hsa-miR-144-3p**	Sig. (2-tailed)	0.012	0.29	0	0	0	0	0	0.001	0	0	0	0	0	0.007
	N	40	37	40	40	40	40	40	40	40	40	40	40	40	40
**hsa-miR-7-5p**	Correlation Coefficient	.380*	0.219	-0.307	-.556**	-.574**	-.449**	-.428**	-.578**	-.487**	-.556**	-.318*	-.464**	-.546**	-.343*
	Sig. (2-tailed)	0.016	0.193	0.054	0	0	0.004	0.006	0	0.001	0	0.045	0.003	0	0.03
	N	40	37	40	40	40	40	40	40	40	40	40	40	40	40
		**hsa-miR-223-3p**	**hsa-miR-24-3p**	**hsa-miR-133b**	**hsa-miR-133a-3p**	**hsa-miR-451a**	**hsa-miR-497-5p**	**hsa-miR-107**	**hsa-miR-205-5p**	**hsa-miR-22-5p**	**hsa-miR-103a-3p**	**hsa-miR-210-3p**	**hsa-miR-199a-5p**	**hsa-miR-144-3p**	**hsa-miR-7-5p**
**Age**	Correlation Coefficient	-0.171	-.376*	0.175	0.176	.371*	0.206	.491**	0.196	0.087	.351*	.378*	0.146	.391*	.380*
	Sig. (2-tailed)	0.291	0.017	0.281	0.276	0.019	0.203	0.001	0.225	0.594	0.026	0.016	0.368	0.012	0.016
	N	40	40	40	40	40	40	40	40	40	40	40	40	40	40
**Pack years**	Correlation Coefficient	0.139	0.05	-0.18	-0.166	-0.234	-0.186	-0.085	-0.067	-0.119	-0.125	0.04	0.014	-0.179	0.219
	Sig. (2-tailed)	0.414	0.769	0.285	0.325	0.163	0.269	0.618	0.692	0.483	0.46	0.813	0.934	0.29	0.193
	N	37	37	37	37	37	37	37	37	37	37	37	37	37	37
**hsa-miR-505-3p**	Correlation Coefficient	.553**	.593**	-.434**	-0.084	-.502**	-0.31	-.508**	-0.233	-0.1	-.498**	-.509**	-0.146	-.566**	-0.307
	Sig. (2-tailed)	0	0	0.005	0.608	0.001	0.051	0.001	0.147	0.538	0.001	0.001	0.37	0	0.054
	N	40	40	40	40	40	40	40	40	40	40	40	40	40	40
**hsa-miR-23b-3p**	Correlation Coefficient	.745**	.793**	-.330*	-0.143	-.625**	-0.113	-.526**	-0.131	-0.131	-.437**	-.458**	-0.234	-.688**	-.556**
	Sig. (2-tailed)	0	0	0.037	0.378	0	0.489	0	0.421	0.422	0.005	0.003	0.147	0	0
	N	40	40	40	40	40	40	40	40	40	40	40	40	40	40

**hsa-miR-361-5p**	Correlation Coefficient	.646**	.816**	-.412**	-0.308	-.610**	-0.233	-.514**	-0.073	-0.276	-.581**	-.494**	-.425**	-.749**	-.574**
	Sig. (2-tailed)	0	0	0.008	0.053	0	0.147	0.001	0.653	0.085	0	0.001	0.006	0	0
	N	40	40	40	40	40	40	40	40	40	40	40	40	40	40
**hsa-miR-27a-3p**	Correlation Coefficient	.755**	.890**	-.515**	-0.099	-.663**	-0.188	-.565**	-0.136	-.317*	-.590**	-.531**	-0.188	-.734**	-.449**
	Sig. (2-tailed)	0	0	0.001	0.545	0	0.246	0	0.402	0.046	0	0	0.246	0	0.004
	N	40	40	40	40	40	40	40	40	40	40	40	40	40	40
**hsa-miR-328-3p**	Correlation Coefficient	.556**	.601**	-.361*	-0.147	-.503**	-0.305	-.523**	-.317*	-0.138	-.542**	-.316*	-0.191	-.549**	-.428**
	Sig. (2-tailed)	0	0	0.022	0.365	0.001	0.055	0.001	0.046	0.397	0	0.047	0.238	0	0.006
	N	40	40	40	40	40	40	40	40	40	40	40	40	40	40
**hsa-let-7b-3p**	Correlation Coefficient	0.187	.496**	-0.151	-0.062	-.496**	-.320*	-.458**	-0.206	-0.182	-.487**	-0.134	-0.288	-.495**	-.578**
	Sig. (2-tailed)	0.249	0.001	0.353	0.702	0.001	0.044	0.003	0.202	0.262	0.001	0.409	0.071	0.001	0
	N	40	40	40	40	40	40	40	40	40	40	40	40	40	40
**hsa-miR-425-3p**	Correlation Coefficient	.592**	.521**	-0.245	-0.2	-.411**	-0.306	-.391*	-0.3	-0.136	-.465**	-0.306	-0.302	-.591**	-.487**
	Sig. (2-tailed)	0	0.001	0.128	0.215	0.008	0.055	0.013	0.06	0.403	0.002	0.055	0.058	0	0.001
	N	40	40	40	40	40	40	40	40	40	40	40	40	40	40
**hsa-miR-197-3p**	Correlation Coefficient	.823**	.726**	-.469**	-0.268	-.610**	-.471**	-.637**	-0.277	-.316*	-.661**	-.434**	-.314*	-.777**	-.556**
	Sig. (2-tailed)	0	0	0.002	0.095	0	0.002	0	0.084	0.047	0	0.005	0.049	0	0
	N	40	40	40	40	40	40	40	40	40	40	40	40	40	40
**hsa-miR-223-5p**	Correlation Coefficient	.688**	.547**	-.330*	-.374*	-.531**	-0.311	-.493**	-.374*	-0.251	-.381*	-.571**	-.351*	-.577**	-.318*
	Sig. (2-tailed)	0	0	0.037	0.018	0	0.051	0.001	0.017	0.119	0.015	0	0.026	0	0.045
	N	40	40	40	40	40	40	40	40	40	40	40	40	40	40

**hsa-miR-191-5p**	Correlation Coefficient	.684**	.606**	-.504**	-0.268	-.679**	-.569**	-.692**	-.533**	-.425**	-.684**	-.543**	-.493**	-.763**	-.464**
	Sig. (2-tailed)	0	0	0.001	0.094	0	0	0	0	0.006	0	0	0.001	0	0.003
	N	40	40	40	40	40	40	40	40	40	40	40	40	40	40
**hsa-miR-23a-3p**	Correlation Coefficient	.673**	.852**	-.321*	-0.08	-.639**	-0.08	-.451**	-0.012	-0.199	-.441**	-.421**	-0.26	-.683**	-.546**
	Sig. (2-tailed)	0	0	0.043	0.626	0	0.624	0.004	0.941	0.217	0.004	0.007	0.105	0	0
	N	40	40	40	40	40	40	40	40	40	40	40	40	40	40
**hsa-miR-423-3p**	Correlation Coefficient	.612**	.404**	-0.26	-0.011	-.421**	-.338*	-.315*	-.407**	-0.217	-0.299	-.360*	-0.224	-.417**	-.343*
	Sig. (2-tailed)	0	0.01	0.105	0.946	0.007	0.033	0.048	0.009	0.178	0.061	0.023	0.165	0.007	0.03
	N	40	40	40	40	40	40	40	40	40	40	40	40	40	40
**hsa-miR-223-3p**	Correlation Coefficient	1	.705**	-.567**	-0.233	-.612**	-.432**	-.659**	-.452**	-0.301	-.635**	-.514**	-0.267	-.782**	-.363*
	Sig. (2-tailed)		0	0	0.148	0	0.005	0	0.003	0.059	0	0.001	0.096	0	0.022
	N	40	40	40	40	40	40	40	40	40	40	40	40	40	40
**hsa-miR-24-3p**	Correlation Coefficient	.705**	1	-.383*	-0.104	-.711**	-0.216	-.586**	-0.172	-0.208	-.619**	-.407**	-0.242	-.789**	-.584**
	Sig. (2-tailed)	0		0.015	0.522	0	0.181	0	0.288	0.198	0	0.009	0.133	0	0
	N	40	40	40	40	40	40	40	40	40	40	40	40	40	40
**hsa-miR-133b**	Correlation Coefficient	-.567**	-.383*	1	.451**	.493**	.514**	.626**	0.288	.397*	.610**	.459**	.450**	.499**	0.21
	Sig. (2-tailed)	0	0.015		0.003	0.001	0.001	0	0.071	0.011	0	0.003	0.004	0.001	0.194
	N	40	40	40	40	40	40	40	40	40	40	40	40	40	40
**hsa-miR-133a-3p**	Correlation Coefficient	-0.233	-0.104	.451**	1	0.291	.471**	.377*	0.186	.359*	.346*	0.232	.598**	0.251	0.16
	Sig. (2-tailed)	0.148	0.522	0.003		0.069	0.002	0.016	0.25	0.023	0.029	0.15	0	0.118	0.325
	N	40	40	40	40	40	40	40	40	40	40	40	40	40	40

**hsa-miR-451a**	Correlation Coefficient	-.612**	-.711**	.493**	0.291	1	.509**	.679**	.353*	.397*	.658**	.347*	.381*	.767**	.430**
	Sig. (2-tailed)	0	0	0.001	0.069		0.001	0	0.026	0.011	0	0.028	0.015	0	0.006
	N	40	40	40	40	40	40	40	40	40	40	40	40	40	40
**hsa-miR-497-5p**	Correlation Coefficient	-.432**	-0.216	.514**	.471**	.509**	1	.572**	.623**	0.274	.570**	0.214	.437**	.450**	.321*
	Sig. (2-tailed)	0.005	0.181	0.001	0.002	0.001		0	0	0.087	0	0.186	0.005	0.004	0.044
	N	40	40	40	40	40	40	40	40	40	40	40	40	40	40
**hsa-miR-107**	Correlation Coefficient	-.659**	-.586**	.626**	.377*	.679**	.572**	1	.493**	.443**	.874**	.603**	.506**	.745**	.371*
	Sig. (2-tailed)	0	0	0	0.016	0	0		0.001	0.004	0	0	0.001	0	0.018
	N	40	40	40	40	40	40	40	40	40	40	40	40	40	40
**hsa-miR-205-5p**	Correlation Coefficient	-.452**	-0.172	0.288	0.186	.353*	.623**	.493**	1	0.196	.429**	.357*	0.219	.339*	0.016
	Sig. (2-tailed)	0.003	0.288	0.071	0.25	0.026	0	0.001		0.225	0.006	0.024	0.175	0.033	0.923
	N	40	40	40	40	40	40	40	40	40	40	40	40	40	40
**hsa-miR-22-5p**	Correlation Coefficient	-0.301	-0.208	.397*	.359*	.397*	0.274	.443**	0.196	1	.460**	0.309	.351*	.494**	0.128
	Sig. (2-tailed)	0.059	0.198	0.011	0.023	0.011	0.087	0.004	0.225		0.003	0.052	0.026	0.001	0.431
	N	40	40	40	40	40	40	40	40	40	40	40	40	40	40
**hsa-miR-103a-3p**	Correlation Coefficient	-.635**	-.619**	.610**	.346*	.658**	.570**	.874**	.429**	.460**	1	.565**	.458**	.803**	.390*
	Sig. (2-tailed)	0	0	0	0.029	0	0	0	0.006	0.003		0	0.003	0	0.013
	N	40	40	40	40	40	40	40	40	40	40	40	40	40	40
**hsa-miR-210-3p**	Correlation Coefficient	-.514**	-.407**	.459**	0.232	.347*	0.214	.603**	.357*	0.309	.565**	1	0.309	.496**	0.278
	Sig. (2-tailed)	0.001	0.009	0.003	0.15	0.028	0.186	0	0.024	0.052	0		0.053	0.001	0.082
	N	40	40	40	40	40	40	40	40	40	40	40	40	40	40

**hsa-miR-199a-5p**	Correlation Coefficient	-0.267	-0.242	.450**	.598**	.381*	.437**	.506**	0.219	.351*	.458**	0.309	1	.376*	.414**
	Sig. (2-tailed)	0.096	0.133	0.004	0	0.015	0.005	0.001	0.175	0.026	0.003	0.053		0.017	0.008
	N	40	40	40	40	40	40	40	40	40	40	40	40	40	40
**hsa-miR-144-3p**	Correlation Coefficient	-.782**	-.789**	.499**	0.251	.767**	.450**	.745**	.339*	.494**	.803**	.496**	.376*	1	.517**
	Sig. (2-tailed)	0	0	0.001	0.118	0	0.004	0	0.033	0.001	0	0.001	0.017		0.001
	N	40	40	40	40	40	40	40	40	40	40	40	40	40	40
**hsa-miR-7-5p**	Correlation Coefficient	-.363*	-.584**	0.21	0.16	.430**	.321*	.371*	0.016	0.128	.390*	0.278	.414**	.517**	1
	Sig. (2-tailed)	0.022	0	0.194	0.325	0.006	0.044	0.018	0.923	0.431	0.013	0.082	0.008	0.001	
	N	40	40	40	40	40	40	40	40	40	40	40	40	40	40

Spearman rank correlation
*P<0.05

Table S5. Correlation analysis between clinical characteristics and identified significantly dysregulated miRNAs for lung cancer participants compared to stable COPD participants.

		Age	Pack years	hsa-miR-361-5p	hsa-miR-27a-3p	hsa-miR-197-3p	hsa-miR-223-5p	hsa-miR-193a-5p	hsa-miR-423-3p	hsa-miR-223-3p	hsa-miR-106b-3p	hsa-miR-133b	hsa-miR-130a-3p	hsa-miR-497-5p	hsa-miR-1260a	hsa-miR-210-3p	hsa-miR-543
Age	Correlation Coefficient	1.000	.332*	0.256	0.286	.434**	0.243	-0.048	0.205	.443**	0.254	-.453**	-.403**	-.472**	-0.117	-0.211	-0.159
	Sig. (2-tailed)		0.048	0.111	0.074	0.005	0.131	0.769	0.204	0.004	0.114	0.003	0.010	0.002	0.473	0.192	0.328
	N	40	36	40	40	40	40	40	40	40	40	40	40	40	40	40	40
Pack years	Correlation Coefficient	.332*	1.000	-0.075	0.050	0.006	0.039	-0.194	0.121	0.130	0.271	-0.236	-0.264	-.423*	-.374*	-0.184	-0.112
	Sig. (2-tailed)	0.048		0.663	0.772	0.974	0.819	0.258	0.481	0.451	0.109	0.166	0.119	0.010	0.024	0.283	0.515
	N	36	36	36	36	36	36	36	36	36	36	36	36	36	36	36	36
hsa-miR-361-5p	Correlation Coefficient	0.256	-0.075	1.000	.867**	.767**	.700**	.646**	.470**	.769**	0.265	-.430**	-.398*	-.325*	-0.187	-.499**	-.344*
	Sig. (2-tailed)	0.111	0.663		0.000	0.000	0.000	0.000	0.002	0.000	0.099	0.006	0.011	0.040	0.247	0.001	0.030
	N	40	36	40	40	40	40	40	40	40	40	40	40	40	40	40	40
hsa-miR-27a-3p	Correlation Coefficient	0.286	0.050	.867**	1.000	.746**	.684**	.693**	.350*	.827**	.320*	-.459**	-.517**	-0.275	-0.201	-.496**	-.380*
	Sig. (2-tailed)	0.074	0.772	0.000		0.000	0.000	0.000	0.027	0.000	0.044	0.003	0.001	0.086	0.213	0.001	0.016

	N	40	36	40	40	40	40	40	40	40	40	40	40	40	40	40	40
hsa-miR-197-3p	Correlation Coefficient	.434**	0.006	.767**	.746**	1.000	.569**	.461**	.474**	.838**	.360*	-.440**	-.367*	-0.309	-0.071	-.421**	-0.281
	Sig. (2-tailed)	0.005	0.974	0.000	0.000		0.000	0.003	0.002	0.000	0.022	0.005	0.020	0.052	0.663	0.007	0.079
	N	40	36	40	40	40	40	40	40	40	40	40	40	40	40	40	40
hsa-miR-223-5p	Correlation Coefficient	0.243	0.039	.700**	.684**	.569**	1.000	.632**	.435**	.594**	.427**	-.328*	-.414**	-.313*	-0.230	-.484**	-.463**
	Sig. (2-tailed)	0.131	0.819	0.000	0.000	0.000		0.000	0.005	0.000	0.006	0.039	0.008	0.049	0.153	0.002	0.003
	N	40	36	40	40	40	40	40	40	40	40	40	40	40	40	40	40
hsa-miR-193a-5p	Correlation Coefficient	-0.048	-0.194	.646**	.693**	.461**	.632**	1.000	0.308	.502**	.316*	-0.224	-.527**	0.002	-0.218	-0.231	-.395*
	Sig. (2-tailed)	0.769	0.258	0.000	0.000	0.003	0.000		0.053	0.001	0.047	0.164	0.000	0.992	0.177	0.152	0.012
	N	40	36	40	40	40	40	40	40	40	40	40	40	40	40	40	40
hsa-miR-423-3p	Correlation Coefficient	0.205	0.121	.470**	.350*	.474**	.435**	0.308	1.000	.507**	.510**	-0.231	-0.296	-0.153	-0.287	-0.280	-0.279
	Sig. (2-tailed)	0.204	0.481	0.002	0.027	0.002	0.005	0.053		0.001	0.001	0.152	0.064	0.346	0.073	0.080	0.081
	N	40	36	40	40	40	40	40	40	40	40	40	40	40	40	40	40
hsa-miR-223-3p	Correlation Coefficient	.443**	0.130	.769**	.827**	.838**	.594**	.502**	.507**	1.000	.459**	-.450**	-.452**	-.446**	-0.271	-.474**	-.487**
	Sig. (2-tailed)	0.004	0.451	0.000	0.000	0.000	0.000	0.001	0.001		0.003	0.004	0.003	0.004	0.091	0.002	0.001
	N	40	36	40	40	40	40	40	40	40	40	40	40	40	40	40	40

hsa-miR-106b-3p	Correlation Coefficient	0.254	0.271	0.265	.320*	.360*	.427**	.316*	.510**	.459**	1.000	-.374*	-.517**	-0.246	-.492**	-.345*	-.478**
	Sig. (2-tailed)	0.114	0.109	0.099	0.044	0.022	0.006	0.047	0.001	0.003		0.018	0.001	0.126	0.001	0.029	0.002
	N	40	36	40	40	40	40	40	40	40	40	40	40	40	40	40	40
hsa-miR-133b	Correlation Coefficient	-.453**	-0.236	-.430**	-.459**	-.440**	-.328*	-0.224	-0.231	-.450**	-.374*	1.000	.555**	.425**	.351*	.535**	0.153
	Sig. (2-tailed)	0.003	0.166	0.006	0.003	0.005	0.039	0.164	0.152	0.004	0.018		0.000	0.006	0.026	0.000	0.347
	N	40	36	40	40	40	40	40	40	40	40	40	40	40	40	40	40
hsa-miR-130a-3p	Correlation Coefficient	-.403**	-0.264	-.398*	-.517**	-.367*	-.414**	-.527**	-0.296	-.452**	-.517**	.555**	1.000	0.170	.460**	.561**	.320*
	Sig. (2-tailed)	0.010	0.119	0.011	0.001	0.020	0.008	0.000	0.064	0.003	0.001	0.000		0.295	0.003	0.000	0.044
	N	40	36	40	40	40	40	40	40	40	40	40	40	40	40	40	40
hsa-miR-497-5p	Correlation Coefficient	-.472**	-.423*	-.325*	-0.275	-0.309	-.313*	0.002	-0.153	-.446**	-0.246	.425**	0.170	1.000	.468**	.396*	.322*
	Sig. (2-tailed)	0.002	0.010	0.040	0.086	0.052	0.049	0.992	0.346	0.004	0.126	0.006	0.295		0.002	0.011	0.043
	N	40	36	40	40	40	40	40	40	40	40	40	40	40	40	40	40
hsa-miR-1260a	Correlation Coefficient	-0.117	-.374*	-0.187	-0.201	-0.071	-0.230	-0.218	-0.287	-0.271	-.492**	.351*	.460**	.468**	1.000	.410**	.446**
	Sig. (2-tailed)	0.473	0.024	0.247	0.213	0.663	0.153	0.177	0.073	0.091	0.001	0.026	0.003	0.002		0.009	0.004
	N	40	36	40	40	40	40	40	40	40	40	40	40	40	40	40	40

hsa-miR-210-3p	Correlation Coefficient	-0.211	-0.184	-.499**	-.496**	-.421**	-.484**	-0.231	-0.280	-.474**	-.345*	.535**	.561**	.396*	.410**	1.000	0.210
	Sig. (2-tailed)	0.192	0.283	0.001	0.001	0.007	0.002	0.152	0.080	0.002	0.029	0.000	0.000	0.011	0.009		0.194
	N	40	36	40	40	40	40	40	40	40	40	40	40	40	40	40	40
hsa-miR-543	Correlation Coefficient	-0.159	-0.112	-.344*	-.380*	-0.281	-.463**	-.395*	-0.279	-.487**	-.478**	0.153	.320*	.322*	.446**	0.210	1.000
	Sig. (2-tailed)	0.328	0.515	0.030	0.016	0.079	0.003	0.012	0.081	0.001	0.002	0.347	0.044	0.043	0.004	0.194	
	N	40	36	40	40	40	40	40	40	40	40	40	40	40	40	40	40

Spearman rank correlation
*P<0.05

References

1. Fernando MR, Jiang C, Krzyzanowski GD, Ryan WL. New evidence that a large proportion of human blood plasma cell-free DNA is localized in exosomes. PloS one. 2017;12(8):e0183915-e.
2. Hassan PA, Rana S, Verma G. Making sense of Brownian motion: colloid characterization by dynamic light scattering. Langmuir. 2015;31(1):3-12.
3. Pritchard CC, Kroh E, Wood B, Arroyo JD, Dougherty KJ, Miyaji MM, et al. Blood cell origin of circulating microRNAs: a cautionary note for cancer biomarker studies. Cancer prevention research (Philadelphia, Pa). 2012;5(3):492-7.
4. Witwer KW, Buzás EI, Bemis LT, Bora A, Lässer C, Lötvall J, et al. Standardization of sample collection, isolation and analysis methods in extracellular vesicle research. Journal of extracellular vesicles. 2013;2:10.3402/jev.v2i0.20360.
5. Andersen CL, Jensen JL, Ørntoft TF. Normalization of real-time quantitative reverse transcription-PCR data: a model-based variance estimation approach to identify genes suited for normalization, applied to bladder and colon cancer data sets. Cancer research. 2004;64(15):5245-50.
6. Schwarzenbach H, da Silva AM, Calin G, Pantel K. Data Normalization Strategies for MicroRNA Quantification. Clin Chem. 2015;61(11):1333-42.
7. Faraldi M, Gomarasca M, Sansoni V, Perego S, Banfi G, Lombardi G. Normalization strategies differently affect circulating miRNA profile associated with the training status. Scientific reports. 2019;9(1):1584.
8. Vlachos IS, Zagganas K, Paraskevopoulou MD, Georgakilas G, Karagkouni D, Vergoulis T, et al. DIANA-miRPath v3.0: deciphering microRNA function with experimental support. Nucleic acids research. 2015;43(W1):W460-W6.

miRCURY LNA miRNA Expression Analysis Report

Hannah O'Farrell

05-26-2021
